# PIF4-mediated thermomorphogenesis relies on its oligomerization ability, not DNA-binding or transactivation activity

**DOI:** 10.21203/rs.3.rs-7294532/v1

**Published:** 2025-09-03

**Authors:** Haibo Xiong, Abhishesh Bajracharya, Ranjeeta Odari, Eden E. Bayer, Alyssa Stoner, Anupa Wasti, Jing Xi, Scott R. Baerson, Yongjian Qiu

**Affiliations:** 1 Department of Biology, University of Mississippi, University, MS 38677, USA; 2 Natural Products Utilization Research Unit, U.S. Department of Agriculture, Agricultural Research Service, University, MS 38677, USA; 3 National Center for Natural Products Research, School of Pharmacy, University of Mississippi, University, MS 38677, USA; 4 These authors contributed equally

**Keywords:** Thermomorphogenesis, Phytochrome-Interacting Factor 4 (PIF4), Basic helix-loop-helix (bHLH), Transcriptional activation domain (TAD), DNA-binding domain (DBD), Intrinsically disordered region (IDR), Phase separation

## Abstract

Plants tailor their architecture to warm ambient temperatures through the central thermosensory transcription factor PHYTOCHROME-INTERACTING FACTOR 4 (PIF4), yet the sequence features that confer this activity remain poorly defined. Here, we combine targeted mutagenesis, phase-separation assays, and transgenic complementation to dissect how structured and disordered regions of PIF4 contribute to its function in thermomorphogenesis. A long N-terminal intrinsically disordered region (IDR) enables PIF4 to form gel-like condensates in vitro and *in planta*. Within this IDR, we identify an acidic transactivation domain (TAD) and an extended basic segment that carries a nuclear-localization signal and the canonical basic motif of the basic helix-loop-helix (bHLH) domain. The basic segment is both necessary and sufficient to drive PIF4 condensate formation, while the TAD merely tunes condensate properties. Strikingly, alanine substitutions that abolish TAD-mediated transactivation, disrupt DNA binding, or greatly reduce phase-separation propensity have no significant effect on thermomorphogenetic hypocotyl elongation. By contrast, replacing three conserved basic residues in the first helix of the HLH domain disrupts PIF4 oligomerization and abolishes thermo-induced hypocotyl growth. We conclude that the ability of PIF4 to oligomerize, rather than to bind DNA or recruit the transcriptional machinery, is the primary determinant of its thermomorphogenetic activity.

## One-sentence summary

Sequence features that define PIF4’s thermomorphogenetic function

## Introduction

Plants react distinctly to different temperature regimes. Growth and development are severely impeded at extreme heat or cold, as cell damage hinders normal physiological processes and energy and resources are redirected from growth to stress tolerance and defense^1,2^. By contrast, within the non-stressful ambient range (≈12–27 °C), moderate warming strongly remodels seedling morphology—hypocotyls, roots, and petioles elongate, reshaping overall architecture in a process known as thermomorphogenesis^3,4^. These changes are driven by multiple thermosensory transcription factors (TTFs), which accumulate and/or activate as temperature rises and regulate genes involved in phytohormone signaling and cell-wall modifications^5,6^.

*Arabidopsis thaliana* TTFs identified to date span multiple gene families, including but not limited to PHYTOCHROME-INTERACTING FACTORs (PIFs), B-BOX proteins (BBXs), Class A1 HEAT SHOCK FACTORs (HSFA1s), TEOSINTE BRANCHED 1/CYCLOIDEA/PCF proteins (TCPs), and BRASSINAZOLE RESISTANT 1 (BZR1)^7–17^. At the hub of their regulatory network is the basic Helix-Loop-Helix (bHLH) transcription factor PIF4, as nearly every other TTF converges on its pathway by modulating at least one aspect of PIF4—its transcription, DNA-binding, transcriptional activity, and protein stability^18^. For example, BZR1 and TCP5 bind the *PIF4* promoter and boost its expression upon temperature elevation^12,13,17^, whereas thermo-induced nuclear accumulation of HSFA1s not only enhances *PIF4* transcription but also stabilizes PIF4 protein^15,16^. PIF4’s pivotal status in thermosensory growth is underscored by its position immediately downstream of the two established thermosensors—phytochrome B (phyB) and the evening-complex component ELF3^19–24^. Multiple BBX proteins, including BBX18, BBX23, BBX24, and BBX25, have been reported to promote PIF4-driven thermomorphogenesis by physically interacting with and antagonizing ELF3^9–11^.

PIF4’s centrality is unsurprising given that the eight-member PIF family (PIF1–PIF8) underpins numerous pathways, including photomorphogenesis, shade avoidance, temperature signaling, and immunity^25–27^. Acting as a signaling hub, PIF4 integrates diverse cues—light, temperature, drought, salinity, and pathogen attack—and thereby mediates growth-defense trade-offs, a role exemplified by its control of leaf senescence^28–36^. Yet, aside from two sequence features shared by all PIFs—an N-terminal active phyB-binding (APB) motif that confers phyB interaction and a C-terminal bHLH domain for DNA binding and oligomerization^37^, the sequence-function landscape of PIF4 remains largely unexplored.

Eukaryotic transcription factors (TFs) are characterized by two core functions: a DNA-binding capacity that recognizes specific sequences (e.g., *cis*-elements in promoters and enhancers) and a transactivation activity that recruits cofactors to modulate transcription^38,39^. Accordingly, TFs are generally organized into a DNA-binding domain (DBD) and one or more effector domains (EDs), such as transcriptional activation domains (TADs) and repression domains (RDs)^40–43^. Many TFs also include auxiliary regions that mediate protein-protein interactions (e.g., oligomerization) and other regulatory processes (e.g., post-translational modifications). These contacts with DNA or protein cofactors arise from both ordered domains, such as DBDs or oligomerization domains, and intrinsically disordered regions (IDRs) that can adopt multiple conformations^44^. Mounting evidence shows that multivalent interactions among IDRs drive phase separation to form biomolecular condensates that facilitate transcription^45^. Notably, many TADs partition with the Mediator coactivator complex into such condensates, linking a TF’s activation potential to the phase-separation propensity of its TAD^46^. Though some DBDs (e.g., zinc finger and homeodomain) allow TFs to bind DNA as monomers, many TFs engage DNA as dimers or higher-order oligomers^47^. This requirement is especially pronounced in the bHLH, bZIP, and MADS-box families, whose noncovalent dimerization or oligomerization is a prerequisite for DNA binding. Emerging evidence suggests that dimerization/oligomerization also contributes to the nuclear condensate formation of several TFs^48,49^.

PIF4 is a typical bHLH TF. It carries a bipartite DBD: a basic stretch rich in arginines that contacts DNA and an HLH module that drives both homo- and hetero-oligomerization^50–52^. Upon warm-temperature cues, PIF4 accumulates in the nucleus and binds promoters of growth-related genes—particularly those governing auxin biosynthesis and signaling, such as *YUC8*, *IAA19*, and *IAA29*^7,53,54^. PIF4 also physically engages several Mediator subunits, including MED14 and MED25, to activate thermo-induced gene expression^55,56^, implying the existence of a functional TAD. In PIF3, a close homolog, the transactivation activity maps to a p53-like TAD featured by a ΦxxΦΦ motif (Φ, hydrophobic residues)^57^. Mutating this motif dramatically disrupts PIF3’s transcriptional activation activity both in yeast and *in planta*, whereas an equivalent mutation in PIF4 only modestly reduces its activity, leaving its precise TAD undefined. Recent work shows that PIF4 can phase separate with JASMONATE ZIM-domain protein 3 (JAZ3), which restricts PIF4’s DNA-binding and transcriptional activation activities^58^. However, whether PIF4 phase separation is indeed crucial for its thermomorphogenetic function and whether the condensate formation relies on an as-yet-unmapped TAD, its oligomeric bHLH core, or both remains unknown.

In this study, we dissect whether and how PIF4’s structured bHLH core and its “dark-matter” IDR contribute to plant thermomorphogenesis. We uncover a long N-terminal IDR that forms gel-like condensates in vitro and *in planta*, yet these assemblies are temperature-insensitive and exhibit limited fluidity. Interestingly, eliminating phase separation—by either mutating the acidic-type TAD or substituting basic residues in the DNA-binding motif—barely impairs PIF4’s ability to restore warm temperature-induced hypocotyl elongation in the *pif4* mutant. By contrast, substituting three basic residues in the first helix of PIF4’s HLH domain abolishes higher-order oligomerization and extinguishes thermomorphogenetic growth. We therefore propose that PIF4 drives thermosensory gene expression primarily through oligomerization and potentially via the long N-terminal IDR, while canonical DNA binding, classical transactivation, and transcriptional condensate formation are largely dispensable.

## Results

### PIF4 forms gel-like condensates that are insensitive to ambient temperatures

**Figure 1.**
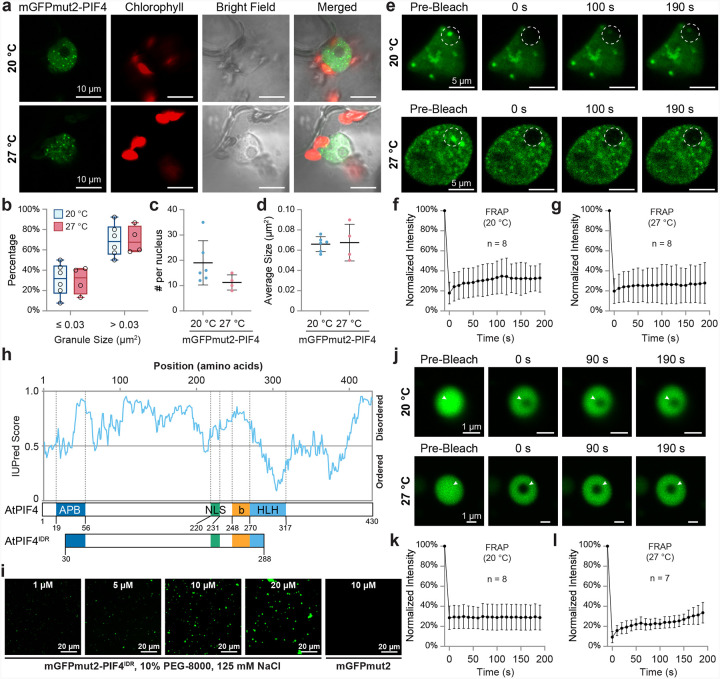
PIF4 forms gel-like condensates via its intrinsically disordered region. **a**, Confocal microscopy images of mGFPmut2-PIF4 speckles in *Nicotiana benthamiana* at 20 °C and 27 °C. **b–d**, Quantitative analysis of mGFPmut2-PIF4 speckles in *N. benthamiana*. **b**, Proportions of smaller (≤ 0.3 μm^2^) and larger (> 0.3 μm^2^) speckles. **c**, Number of granules per nucleus. **d**, Average granule size. No significant differences were observed between granules formed at 20 °C and 27 °C (Student’s t-test, n ≥ 4). **e**, Representative confocal microscopy images illustrating fluorescence recovery after photobleaching (FRAP) of mGFPmut2-PIF4 speckles in *N. benthamiana* at 20 °C and 27 °C. White circles indicate the bleached regions. **f–g**, Quantification of fluorescence intensity at the bleached regions before and after FRAP at 20 °C (**f**) and 27 °C (**g**). Data were recorded and analyzed from at least 8 nuclei. **h**, IUPred prediction of the intrinsically disordered region (IDR) in *Arabidopsis thaliana* PIF4. A long IDR spanning amino acids 30–288 was identified. Abbreviations: APB, active phyB-binding motif; NLS, nuclear localization signal; bHLH, basic helix-loop-helix domain. **i**, In vitro phase separation assay of recombinant mGFPmut2-PIF4^IDR^. Proteins at varying concentrations (1, 5, 10, and 20 μM) were incubated in 10% PEG-8000 with 125 mM NaCl. **j**, Confocal microscopy images of FRAP on mGFPmut2-PIF4^IDR^ speckles in vitro at 20 °C and 27 °C. White arrowheads indicate the bleached regions. **k–l**, Quantification of fluorescence intensity at the bleached regions before and after FRAP at 20 °C (**k**) and 27 °C (**l**). Data were recorded and analyzed from 7–8 speckles.

PIF4 was reported to form nuclear speckles in plants^58,59^. Some key thermosensory transcription factors, such as HSFA1a, undergo phase transition to liquid droplets as a function of temperature^60^. To investigate whether the formation of PIF4 nuclear speckles is affected by ambient temperatures, we expressed mGFPmut2-tagged PIF4 in *Nicotiana benthamiana* leaves through Agro-infiltration and compared nuclear speckle formation under cooler (20 °C) and warmer temperatures (27 °C). At both temperatures, mGFPmut2-PIF4 proteins were localized to distinct nuclear speckles and nucleoplasm (Fig. 1a). The number and size of these granules were comparable between 20 °C and 27 °C (Fig. 1b–d). We further applied fluorescence recovery after photobleaching (FRAP) to compare the liquidity of mGFPmut2-PIF4 nuclear speckles in tobacco leaves. The fluorescent signal in the photobleached region marginally recovered at both temperatures (Fig. 1e–g), suggesting an ambient temperature-insensitive, gel-like structure of mGFPmut2-PIF4 condensates.

Unlike HSFA1a, ELF3, and PIF7^24,59,61^, which possess prion-like low complexity domains (PrLDs), PIF4 does not display a high-confidence PrLD score in PLAAC^58^. We hypothesize that PIF4 harbors other IDRs that may contribute to its gel-like phase separation in plants. Indeed, IUPred3^62^ predicts a long IDR in the N-terminus of PIF4 protein (Fig. 1h). This PIF4^IDR^, spanning from 30 to 288 amino acids, includes a long unstructured region (57–219 a.a.) and also some known domains/motifs, such as most of the Active Phytochrome B-binding (APB) domain, the nuclear localization signal (NLS), the basic DNA-binding region, and the first helix in the HLH dimerization/tetramerization domain^51^. We fused PIF4^IDR^ with an N-terminal mGFPmut2 and tested its phase separation ability in vitro. In the presence of the macromolecular crowder PEG-8000 (10%), the recombinant mGFPmut2-PIF4^IDR^ protein phase separated into near-spherical condensates at a minimum concentration of 1 μM, and the granule size increased as a function of the protein concentration (Fig. 1i). We further performed FRAP on these in vitro-formed mGFPmut2-PIF4^IDR^ condensates and discovered that they displayed poor liquidity at both 20 °C and 27 °C (Fig. 1j–l). These data suggest that PIF4 can form gel-like condensates both in vitro and in vivo, and its phase separation ability may not be affected within the ambient temperature range.

### PIF4 possesses an N-terminal TAD

Intrinsically disordered TADs of transcriptional activators have been reported to play a crucial role in transcriptional condensate formation and subsequent gene expression^46,63^. However, a recent study contended that TAD-mediated multivalent interactions, rather than the phase separation capacity of a transcription factor, are essential for transcriptional activation^64^. We set out to investigate the relationship between the PIF4 TAD, its phase separation propensity, and its function in thermoresponsive gene expression.

While the TAD of PIF3, the prototypical PIF protein, has been extensively characterized^57,65^, the precise sequence and critical features of the PIF4 TAD remain unknown. We first mapped the PIF4 TAD using a yeast transactivation system. Three fragments, PIF4A (1–78 a.a.), PIF4B (79–242 a.a.), and PIF4C (243–430 a.a.), were each fused to the GAL4 DBD. Only the N-terminal 78 a.a. displayed strong transactivation activity in yeast (Supplementary Fig. 1a). Further dissection of the PIF4A fragment revealed no transactivation activity (Supplementary Fig. 1b).

**Figure 2.**
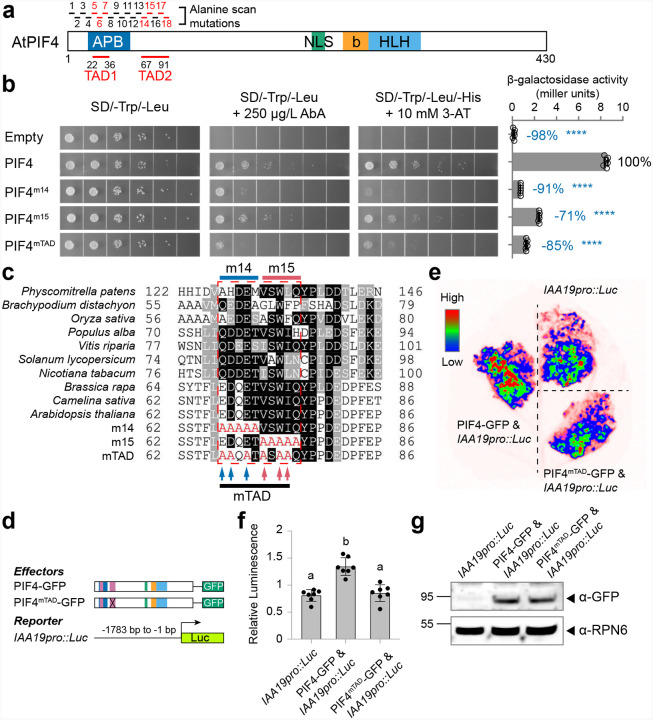
Identification of the transactivation domain in PIF4 N-terminus. **a**, Schematic representation of the PIF4 domain structure. Alanine-scanning mutants (m1–m18) were generated and analyzed in Supplementary Figure S1 to identify regions critical for transactivation activity. Mutants exhibiting reduced transcriptional activity in yeast, as well as the two transactivation domains (TADs) identified, are highlighted in red. Abbreviations: APB, active phyB-binding motif; NLS, nuclear localization signal; bHLH, basic helix-loop-helix domain. **b**, Alanine-scanning mutagenesis of PIF4 to determine residues essential for TAD activity. Transcriptional activity was assessed using three yeast reporter systems: Aureobasidin A (AbA) resistance, HIS3 (-His), and LacZ (β-galactosidase liquid assay). The activity of wild-type and mutant PIF4 versions is shown. **c**, Sequence alignment of the TAD2 region across PIF4 homologs in various land plants. Arrows indicate mutated residues in the PIF4^mTAD^ mutant. Species include *Arabidopsis thaliana* (NP_001323426.1), *Camelina sativa* (XP_010506153.1), *Brassica rapa* (XP_018513637.1), *Nicotiana tabacum* (XP_016471316.1), *Solanum lycopersicum* (XP_019070178.1), *Vitis riparia* (XP_034702447.1), *Populus alba* (XP_034915052.1), *Oryza sativa* (XP_015618074.1), *Brachypodium distachyon* (KQK14025.1), and *Physcomitrella patens* (Pp3c1_38820V3.1.p). **d**, Diagram of the reporter and effector constructs used in the tobacco luciferase assay. **e**, Representative tobacco leaf image showing luciferase activity. **f**, Quantification of relative luminescence from the luciferase assay. Statistical significance was determined using one-way ANOVA (n = 7, p < 0.0001), with different letters indicating significantly different groups. **g**, Immunoblot analysis showing comparable expression levels of PIF4-GFP and PIF4^mTAD^-GFP in tobacco leaves. RPN6 was used as a loading control.

To identify key residues responsible for PIF4’s transactivation activity in the N-terminal region, we conducted alanine scanning mutagenesis across the first 91 a.a. of the PIF4 protein. Beginning with the second residue, we generated 18 mutants (m1–m18) by substituting five consecutive residues with alanines and tested their impact on transactivation activity in yeast. Using three reporters (AUR1-C, HIS3, and LacZ), we identified two regions—m5 to m7 and m14 to m18—that showed a marked reduction in PIF4’s transactivation activity, which we named TAD1 and TAD2, respectively (Supplementary Fig. 1c and Fig. 2a). TAD1 overlaps with the APB domain, and several mutations (e.g., E29V and W32A) are known to disrupt phyB interactions^65^. In contrast, mutations in TAD2, such as W74A, F84A, F89A, and FF93–94AA, had little or no effect on phyB binding^65^.

We were particularly intrigued by m14 (67–71 a.a.) and m15 (72–76 a.a.), which overlap with the p53-like ΦxxΦΦ motif identified in the PIF3 TAD (Supplementary Fig. 1c and Fig. 2b)^57^. The m14 region contains three acidic residues, two of which (D68 and E70) are highly conserved among land plants, and the m15 region harbors three conserved hydrophobic residues (V72, W74, and I75) (Fig. 2c). However, substituting each hydrophobic residue in m15 did not affect PIF4’s transactivation activity in yeast (Supplementary Fig. 1d). Therefore, we generated a mutated TAD variant (mTAD) by substituting the three acidic residues in m14 and the three hydrophobic residues in m15 with alanines (Fig. 2c). Similar to the m14 and m15 mutants, the mTAD variant drastically reduced PIF4’s transactivation activity in yeast (Fig. 2b). We also assessed its effect on PIF4 function *in planta* by co-expressing the PIF4 protein with a firefly luciferase reporter driven by the *IAA19* promoter—a well-known PIF4 target. While wild-type PIF4 significantly boosted luciferase expression, PIF4^mTAD^ failed to do so when expressed at the same level as the wild-type protein (Fig. 2d–g). Consequently, we chose to use the mTAD variant to further explore how the PIF4 TAD contributes to phase separation and gene activation in plants.

### PIF4 TAD mutations modestly affect PIF4 phase separation and its transcriptional activity in thermomorphogenesis

**Figure 3.**
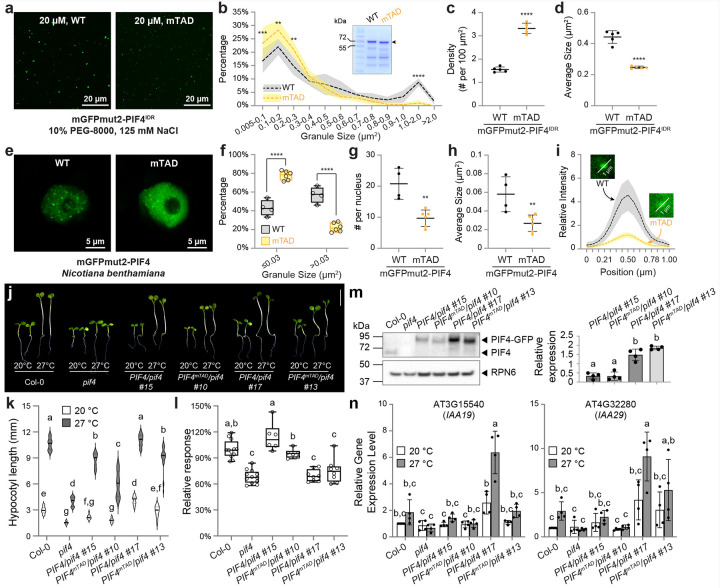
The PIF4 TAD is largely dispensable for thermomorphogenesis. **a**, In vitro phase separation assay of the wild-type and mTAD mutant versions of mGFPmut2-PIF4^IDR^ recombinant proteins. Proteins (20 μM) were incubated in 10% PEG-8000 with 125 mM NaCl. The Coomassie blue staining inside **b** shows protein quality. **b**, Size distribution of mGFPmut2-PIF4^IDR^ granules. Wild-type and mTAD mutant granules were binned into 12 size categories, with the mean percentage for each size bin represented by black (wild-type) or orange (mTAD) lines, and the standard deviation (SD) shown as shaded areas. Statistical analysis was performed using two-way ANOVA (n = 5; **, p < 0.01; ***, p < 0.001; ****, p < 0.0001). **c**, Comparison of granule density between wild-type and mTAD versions of mGFPmut2-PIF4^IDR^. **d**, Average granule sizes of wild-type and mTAD versions of mGFPmut2-PIF4^IDR^. Statistical significance was assessed using a Student’s t-test (n = 5; ****, p < 0.0001). **e**, Confocal microscopy images of wild-type and mTAD versions of mGFPmut2-PIF4 speckles in *N. benthamiana*. **f–i**, Quantitative analysis of mGFPmut2-PIF4 speckles in *N. benthamiana*. **f**, Proportions of smaller (≤ 0.03 μm^2^) and larger (> 0.03 μm^2^) speckles. **g**, Number of granules per nucleus. **h**, Average granule size. **i**, Signal intensity distribution of mGFPmut2-PIF4 speckles. Intensity profiles within a 1-μm range centered on the speckles were compared between wild-type and mTAD mutants. Black (wild-type) and orange (mTAD) lines represent mean relative intensity, with shaded regions indicating the 95% confidence interval (CI). Representative images of wild-type and mTAD speckles are shown. **j**, Representative images of 4-day-old seedlings of various genotypes grown under continuous red light (Rc, 50 μmol m^−2^ s^−1^) at 20 °C and 27 °C. Genotypes include wild-type (Col-0), *pif4*-*2* (*pif4*), two lines expressing *PIF4pro::PIF4*-*mGFPmut2*-^*6*^*His* in the *pif4*-*2* background (*PIF4/pif4* #15 & #17), and two lines expressing *PIF4pro::PIF4*^*mTAD*^-*mGFPmut2*-^*6*^*His* in the *pif4*-*2* background (*PIF4*^*mTAD*^*/pif4* #10 & #13). **k**, Hypocotyl length measurements of seedlings shown in **j**. White and gray violin plots represent data from 20 °C and 27 °C, respectively. Solid lines denote the median, and dotted lines indicate the first and third quartiles. Different letters indicate statistically significant differences in absolute hypocotyl lengths (two-way ANOVA, n ≥ 20, p < 0.01). **l**, Relative thermal response of hypocotyl elongation among the genotypes in **j**. Box plots show the median (center line), first and third quartiles (box limits), and whiskers (minimum and maximum values). Relative response is calculated as the ratio of hypocotyl elongation at 27 °C to 20 °C, normalized to Col-0 (set to 100%). Different letters indicate significant differences between genotypes (one-way ANOVA, n ≥ 6, p < 0.05). **m**, Immunoblot analysis of PIF4 protein levels in the genotypes in **j**. Seedlings were grown at 20 °C and then transferred to 27 °C for 6 hours under Rc. RPN6 was used as a loading control, and relative PIF4 levels were quantified from four biological replicates. Statistical differences were determined by one-way ANOVA (n = 4, p < 0.0001). **n**, RT-qPCR analysis of thermo-induced, growth-promoting gene expression levels in the seedlings shown in **j**. Seedlings were grown at 20 °C and then transferred to 27 °C or maintained at 20 °C for 6 hours under Rc. Data was shown from four biological replicates. Different letters indicate statistically significant differences in absolute hypocotyl lengths (two-way ANOVA, n = 4, p < 0.05).

We first compared the biophysical properties of condensates formed by wild-type PIF4 and its mTAD variant. When the proteins were expressed as mGFPmut2 fusions and incubated with 10% PEG-8000, PIF4^mTAD^ still underwent phase separation (Fig. 3a). Nevertheless, mGFPmut2–PIF4^mTAD^ produced denser condensates with a smaller mean area, owing to a higher proportion of small granules (≤ 0.3 μm^2^) and a scarcity of larger ones (≥ 1.0 μm^2^) compared with the wild-type protein (Fig. 3b–d). A similar pattern emerged in vivo: in tobacco nuclei, the phase-separation propensity of PIF4^mTAD^, expressed as an mGFPmut2 fusion, was diminished (Fig. 3e). Both the total number and the average size of nuclear speckles decreased, with PIF4^mTAD^ generating more small and fewer large speckles (Fig. 3f–h). The mutation also heightened the diffuse nucleoplasmic signal (Fig. 3i). Collectively, substitutions in the m14/m15 region partially compromise the phase-separation capacity of PIF4 both in vitro and in vivo.

We further investigated whether the attenuated transactivation activity (Fig. 2) and reduced phase-separation propensity (Fig. 3a–i) of the mTAD variant would translate into impaired thermomorphogenetic growth in *Arabidopsis*. Accordingly, we generated transgenic lines expressing either wild-type or mTAD PIF4, each with a C-terminal mGFPmut2-^6^His fusion driven by the native *PIF4* promoter in a *pif4* mutant (SAIL_1288_E07) background. We obtained six *PIF4pro::PIF4/pif4* and eight *PIF4pro::PIF4*^*mTAD*^*/pif4* single-insertion lines, which displayed a range of hypocotyl lengths at 20 °C and 27 °C (Supplementary Fig. 2a). Because hypocotyl length correlates with *PIF4* expression, we plotted each line’s absolute hypocotyl length at 27 °C against its relative *PIF4* transcript level (Col-0 = 1) and performed nonlinear regression to compare wild-type and mTAD datasets (Supplementary Fig. 2b). At equivalent expression levels, *PIF4pro::PIF4*^*mTAD*^*/pif4* seedlings exhibited slightly shorter hypocotyls than their wild-type counterparts.

To refine this analysis, we selected two pairs of transgenic lines with comparable *PIF4* transcript levels (Supplementary Fig. 2b and Fig. 3j). In both pairs, PIF4 protein abundance at 27 °C was similar between the mTAD and wild-type lines (Fig. 3m). Nevertheless, thermomorphogenetic hypocotyl elongation was partially compromised in the mTAD lines under both red and white light (Fig. 3j–l and Supplementary Fig. 2c,d). Consistent with the modest hypocotyl shortening, thermo-induced expression of the PIF4 targets *IAA19* and *IAA29* trended lower in the mTAD lines, but only the reduction in *IAA19* level within the higher-PIF4 line pair (*PIF4/pif4* #17 and *PIF4*^*mTAD*^*/pif4* #13) reached statistical significance, whereas changes in the lower-PIF4 pair (*PIF4/pif4* #15 and *PIF4*^*mTAD*^*/pif4* #10) and those in *IAA29* expression across both pairs were not significant (Fig. 3n). Although these data demonstrate that the mTAD substitution diminishes PIF4 function during thermomorphogenesis, the reduction *in planta* is relatively modest, especially given that TAD activity was nearly abolished in yeast (Fig. 2b).

### Acidic and hydrophobic residues in PIF4 TAD are crucial for its phase separation capacity

The observation that the TAD of PIF4—a pivotal thermosensory transcriptional activator—is dispensable for thermomorphogenesis is unexpected. Given the long-recognized length-dependent modularity of TADs^66,67^, we asked whether the six alanine substitutions in PIF4^mTAD^ were simply insufficient to abolish its transactivation capacity *in planta*. Notably, the acidic and hydrophobic character of the PIF4 TAD mirrors that of classical acidic TADs from plant, yeast, and human activators^42,68,69^. According to the recently proposed “Acidic Exposure” model, acidic residues alternate with hydrophobic residues within an acidic TAD, and electrostatic repulsion among the acidic side chains exposes the hydrophobic residues to solvent, allowing co-activator engagement^68,69^. We therefore re-evaluated the PIF4 TAD with the improved mechanistic predictor of acidic TADs that tallies four charged residues (D, E, R, K) and three hydrophobic residues (W, F, L)^70^.

**Figure 4.**
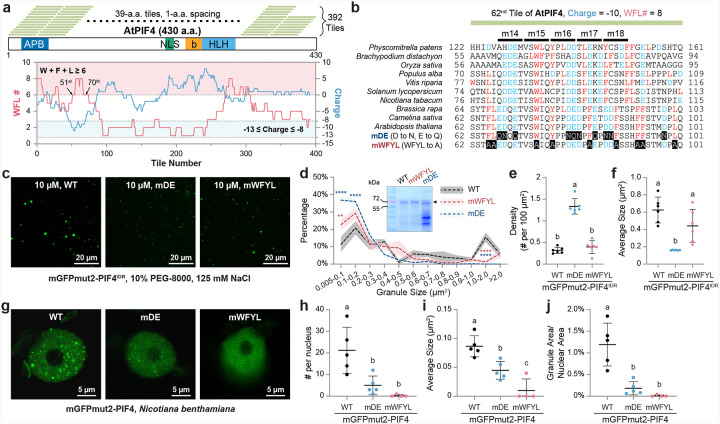
Acidic and hydrophobic residues in the PIF4 TAD are essential for phase separation. **a**, Schematic representation of a computational analysis dividing *Arabidopsis thaliana* PIF4 (AtPIF4) into overlapping 39-amino-acid (a.a.) tiles, spaced by 1 a.a. A total of 392 tiles were analyzed for hydrophobic (W, F, L) and acidic (D, E) residue content. Regions meeting the criteria of an acidic transactivation domain, as predicted by Kotha and Staller (2023)^70^, are shaded red and blue. Criteria include W+F+L ≥ 6 and −13 ≤ Charge ≤ −8. **b**, Sequence alignment of PIF4 homologs, highlighting conserved acidic (blue) and hydrophobic (red) residues. Missense mutations were introduced into AtPIF4 to create two mutant versions: mDE (D mutated to N and E mutated to Q) and mWFYL (hydrophobic residues mutated to alanines). **c**, In vitro phase separation assays of the wild-type, mDE, and mWFYL versions of mGFPmut2-PIF4^IDR^. Recombinant proteins (20 μM) were incubated in 10% PEG-8000 with 125 mM NaCl. The Coomassie blue staining inside **d** shows protein quality. **d**, Size distribution of mGFPmut2-PIF4^IDR^ granules. Mean percentages of granules within 12 size bins are shown for wild-type (black), mDE (dark blue), and mWFYL (dark red). Shaded regions (gray, light blue, light red) indicate the standard error of the mean (SEM). Significant differences between mutants and wild-type were analyzed using two-way ANOVA (n = 6; **, p < 0.01; ****, p < 0.0001). **e**, Comparison of granule densities between wild-type and mutant versions of mGFPmut2-PIF4^IDR^. **f**, Average granule size for wild-type and mutant versions of mGFPmut2-PIF4^IDR^. Statistical significance was determined using one-way ANOVA (n = 6; p < 0.01). **g**, Confocal microscopy images of wild-type, mDE, and mWFYL versions of mGFPmut2-PIF4 speckles in *N. benthamiana*. **h–j**, Quantitative analysis of mGFPmut2-PIF4 speckles in *N. benthamiana*. **h**, Number of granules per nucleus. **i**, Average granule size. **j**, Percentage of granule area relative to nuclear area. Statistical significance was determined using one-way ANOVA (n ≥ 4; p < 0.05).

We dissected PIF4 into 39-amino-acid sliding windows, stepped by a single residue (Fig. 4a). For each of the 392 tiles, we summed the counts of W, F, and L and calculated the net charge (D or E = −1; R or K = +1). Applying the predictor, we found that tiles 51–70, spanning residues 51–109, met both criteria—W + F + L ≥ 6 and −13 ≤ charge ≤ −8. Within this acidic TAD, acidic residues (D and E) are interleaved with bulky hydrophobics (W, F, Y, L) (Fig. 4b). Analyses of PIF4 homologues from other land plants revealed the same architectural motif despite low overall sequence identity (Supplementary Fig. 3 and Fig. 4b).

To test the functional contribution of these residues, we created two mutants: mDE, in which six Ds and four Es were replaced by Ns and Qs, respectively; and mWFYL, in which nine bulky hydrophobic residues were substituted by As (Fig. 4b). Both variants curtailed PIF4^IDR^ phase separation in vitro, yielding more small granules (≤ 0.2 μm^2^) and fewer very large granules (> 1 μm^2^); the reduction was stronger in mDE (Fig. 4c,d). Intriguingly, the overall granule density and mean size of mWFYL granules still resembled those of the wild-type protein (Fig. 4e,f). In vivo, however, both mDE and mWFYL almost completely eliminated PIF4 nuclear speckle formation in *N. benthamiana*, with mWFYL producing a more severe phenotype (Fig. 4g–j). These data suggest that both acidic and hydrophobic residues in PIF4 TAD are crucial for its phase separation propensity.

### The acidic and hydrophobic residues of the PIF4 TAD have distinct roles in thermomorphogenesis

**Figure 5.**
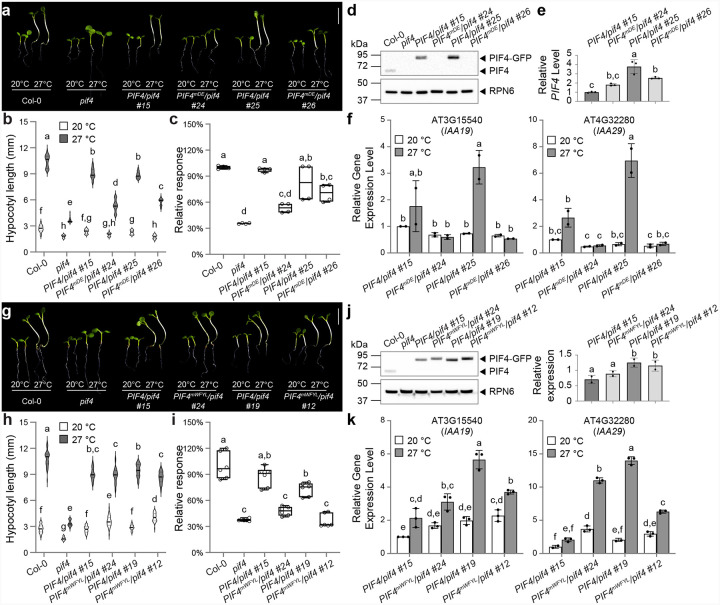
Acidic—but not hydrophobic—TAD residues are crucial for PIF4 and thermomorphogenesis. **a**, Representative seedling images. Wild-type (Col-0), *pif4*-*2* (*pif4*), two lines of *PIF4*-*pro::PIF4*-*mGFPmut2*-^*6*^*His/pif4*-*2* (*PIF4/pif4* #15 & #25), and two lines of *PIF4pro::PIF4*^*mDE*^-*mGFPmut2*-^*6*^*His/pif4*-*2* (*PIF4*^*mDE*^*/pif4* #24 & #26) were grown for 4 days in continuous red (Rc) light (50 μmol m^−2^ s^−1^) at 20 °C and 27 °C. **b**, Hypocotyl length measurements of seedlings in **a**. The white and grey violin plots represent hypocotyl length measurements at 20 °C and 27 °C, respectively. The elements of violin plots are as follows: solid line, median; lower dotted line, first quartile; upper dotted line, third quartile. Different letters denote significant statistical differences between the absolute hypocotyl length of each line (two-way ANOVA, n ≥ 17, p < 0.01). **c**, Comparison of the relative thermal response among the seedlings in **a**. The elements of box plots are as follows: center line, median; box limits, first and third quartiles; whiskers, minimum and maximum values; points, all data points. The relative response is defined as the relative hypocotyl response to 27°C of a mutant compared with that of Col-0 (which is set at 100%). Different letters denote significant statistical differences between the relative response of each genotype (one-way ANOVA, n = 4, p < 0.05). **d**, Immunoblots showing PIF4 protein levels in transgenic lines in **a**. Seedlings were grown for 4 days in 50 μmol m^−2^ s^−1^ Rc at 20 °C and treated at 27 °C for 6 hours under the same light conditions. RPN6 was used as an internal control. **e**, RT-qPCR results showing the relative *PIF4* transcript levels at 27 °C. Data was shown from three technical replicates. Different letters denote significant statistical differences (one-way ANOVA, n = 3, p < 0.05). **f**, RT-qPCR analysis of thermo-induced, growth-promoting gene expression levels. Seedlings were grown at 20 °C and then transferred to 27 °C or maintained at 20 °C for 6 hours under Rc. Data was shown from two biological replicates. Different letters denote significant statistical differences (two-way ANOVA, n = 2, p < 0.05). **g**, Representative seedling images. Col-0, *pif4*, *PIF4/pif4* #15 & #19, and two lines of *PIF4pro::PIF4*^*mWFYL*^-*mGFPmut2*-^*6*^*His/pif4*-*2* (*PIF4*^*mWFYL*^*/pif4* #24 & #12) were grown for 4 days in continuous red (Rc) light (50 μmol m^−2^ s^−1^) at 20 °C and 27 °C. **h**, Hypocotyl length measurements of seedlings in **g**. Different letters denote significant statistical differences between the absolute hypocotyl length of each line (two-way ANOVA, n ≥ 30, p < 0.05). **i**, Comparison of the relative thermal response among the seedlings in **g**. Different letters denote significant statistical differences between the relative response of each genotype (one-way ANOVA, n = 6, p < 0.001). **j**, Immunoblots showing PIF4 protein levels in transgenic lines in **g**. Seedlings were grown for 4 days in 50 μmol m^−2^ s^−1^ Rc at 20 °C and treated at 27 °C for 6 hours under the same light conditions. RPN6 was used as an internal control for normalization. The relative PIF4 protein level was calculated based on two immunoblots using two biological replicates. Different letters denote significant statistical differences (one-way ANOVA, n = 2, p < 0.05). **k**, RT-qPCR analysis of thermo-induced, growth-promoting gene expression levels. Seedlings were grown at 20 °C and then transferred to 27 °C or maintained at 20 °C for 6 hours under Rc. Data was shown from three technical replicates. Different letters denote significant statistical differences (one-way ANOVA, n = 3, p < 0.05).

The strong reduction of nuclear speckles in the mDE and mWFYL variants (Fig. 4) made these two mutants ideal for testing how PIF4 phase-separation capacity relates to its role in thermomorphogenesis. We therefore created single-insertion *PIF4pro::PIF4*^*mDE*^ and *PIF4pro::PIF4*^*mWFYL*^ lines in the *pif4* mutant background and compared their thermo-responsive phenotype with *PIF4pro::PIF4* controls (Fig. 5).

Surprisingly, PIF4^mDE^-mGFPmut2 protein was barely detectable in *PIF4pro::PIF4*^*mDE*^ lines #24 and #26 (Fig. 5d). However, both lines accumulated *PIF4* transcripts at levels between those in *PIF4pro::PIF4* lines #15 and #25 (Fig. 5e), indicating that acidic residues in the PIF4 TAD are required for its protein stability. As expected, both *PIF4pro::PIF4*^*mDE*^ lines failed to rescue the *pif4* thermomorphogenetic defects as their hypocotyls remained as short as those of the *pif4* mutant at 27 °C (Fig. 5a–c). Consistently, thermo-induced expression of *IAA19* and *IAA29* was still suppressed in these *PIF4pro::PIF4*^*mDE*^ lines (Fig. 5f). Because the PIF4^mDE^-mGFPmut2 protein is unstable, these data do not allow us to conclude whether acidic residues are directly required for PIF4’s transactivation activity in thermomorphogenesis.

We next examined the mWFYL mutant, which showed a stronger *in*-*planta* defect in phase separation. Two single-insertion *PIF4pro::PIF4*^*mWFYL*^ lines, #24 and #12, accumulated protein amounts comparable to *PIF4pro::PIF4* lines #15 and #19, respectively (Fig. 5j). Surprisingly, neither *PIF4pro::PIF4*^*mWFYL*^ line showed any defects in thermomorphogenesis—both had similar hypocotyl length as their wild-type counterparts at 27 °C (Fig. 5g–i). Their overall thermo-induced elongation ratios, however, were lower than those of the corresponding wild-type lines (Fig. 5i), largely owing to their longer hypocotyls at 20 °C (Fig. 5h). Once again, thermo-responsive transcription of PIF4 target genes showed a mixed pattern (Fig. 5k). In the higher-expression pair, they were significantly lower in *PIF4pro::PIF4*^*mWFYL*^ line #12 than *PIF4pro::PIF4* line #19; whereas in the lower-expression pair, *IAA19* and *IAA29* levels in *PIF4pro::PIF4*^*mWFYL*^ line #24 were similar to, or even higher than, those in *PIF4pro::PIF4* line #15 (Fig. 5k). Thus, although hydrophobic residues are important for PIF4 phase-separation propensity, they appear largely dispensable for the thermosensory activation of genes driving thermomorphogenetic hypocotyl elongation.

### The basic region in PIF4 IDR is essential for its phase separation capacity

Collectively, our TAD-mutant analyses suggest that PIF4’s phase-separation capacity, as governed by the TAD, is largely uncoupled from its transcriptional output and thermomorphogenetic function. To probe which portion of the IDR (30–288 a.a.) drives PIF4 condensate formation, we extended our focus beyond the TAD itself (51–109 a.a.), which constitutes <25 % of the IDR and is followed by an NLS as well as the basic motif and first helix of the HLH domain. The key question is whether the TAD initiates condensate nucleation or merely modulates assembly orchestrated elsewhere in the IDR. We therefore set out to identify the minimal segment that is both necessary and sufficient for PIF4 condensate formation.

We first performed in vitro phase-separation assays using a series of N- and C-terminal truncations of PIF4^IDR^ (Supplementary Fig. 4a). Remarkably, the extreme C-terminus of PIF4^IDR^ (244–288 a.a.) was indispensable for condensate formation, whereas the N-terminal segment containing the TAD (N0–N4) failed to generate droplets in vitro (Supplementary Fig. 4b,c). By contrast, every C-terminal fragment (C1–C4) lacking the TAD readily produced speckles comparable to those formed by the full-length PIF4^IDR^ (Supplementary Fig. 4b,c). These observations indicate that the TAD functions primarily as a regulator of PIF4 phase separation and is insufficient on its own to drive condensate assembly. Moreover, the C4 segment (217–288 a.a.) is both necessary and sufficient to promote phase separation.

**Figure 6.**
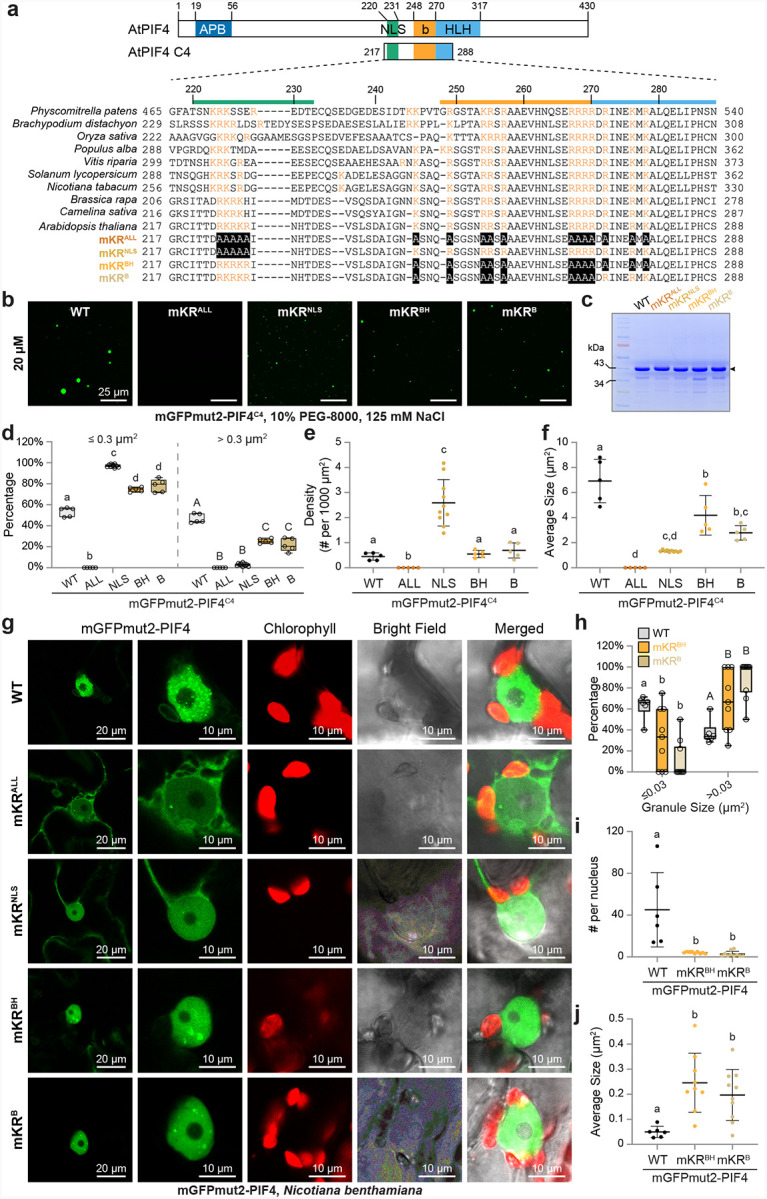
Basic residues in PIF4 IDR are essential for phase separation. **a**, Sequence alignment of PIF4 homologs, highlighting all the basic residues (orange). Missense mutations were introduced into AtPIF4 to create four mutant versions—mKR^ALL^, mKR^NLS^, mKR^BH^, and mKR^B^—in which basic residues were substituted by alanines. **b**, In vitro phase separation assays of the wild-type and mKR versions of mGFPmut2-PIF4^C4^. Recombinant proteins (20 μM) were incubated in 10% PEG-8000 with 125 mM NaCl. **c**, Coomassie blue staining showing the recombinant protein quality used in phase separation assays. **d–f**, Quantitative analysis of the wild-type and mKR versions of mGFPmut2-PIF4^C4^ condensates in vitro. **d**, Proportions of smaller (≤ 0.3 μm^2^) and larger (> 0.3 μm^2^) granules. **e**, Granule densities. **f**, Average granule size. Statistical significance was determined using one-way ANOVA (n ≥ 5; p < 0.01). **g**, Confocal microscopy images of the wild-type and mKR versions of mGFPmut2-PIF4 speckles in *N. benthamiana*. **h–j**, Quantitative analysis of mGFPmut2-PIF4 speckles in *N. benthamiana*. **h**, Proportions of smaller (≤ 0.03 μm^2^) and larger (> 0.03 μm^2^) speckles. **i**, Number of granules per nucleus. **j**, Average granule size. Statistical significance was determined using one-way ANOVA (n ≥ 6; p < 0.05).

The C4 segment harbors three defined structural elements: an NLS peptide, the basic DNA-binding motif, and the first helix of the HLH dimerization/tetramerization domain (Fig. 6a). Although the basic motif and its adjoining helix are highly conserved across land plants, the upstream sequence is markedly diverse (Fig. 6a). Amino-acid composition analysis of the C4 segment confirmed an unusually high proportion of basic residues—arginine plus lysine together account for 20–25%, whereas aromatic residues (W, Y, F) are virtually absent (Fig. 6a; Supplementary Fig. 5). Arginine is known to promote phase separation through cation–π interactions with aromatic residues; recent studies further indicate that its guanidinium group can engage in π–π stacking interactions that promote phase separation^71,72^. We therefore hypothesized that the elevated arginine content in C4 is critical for PIF4’s propensity to phase-separate.

To test this idea, we generated four alanine-substitution variants of C4: mKR^ALL^ (all 17 basic residues), mKR^NLS^ (5 basic residues within the NLS), mKR^BH^ (12 basic residues spanning the basic and helix regions), and mKR^B^ (9 residues confined to the basic motif) (Fig. 6a). These KR substitutions impaired mGFPmut2-PIF4^C4^ phase separation in a dose-dependent manner—mKR^NLS^, mKR^BH^, and mKR^B^ modestly reduced droplet size, whereas mKR^ALL^ completely abolished condensate formation (Fig. 6b–f). Next, we evaluated the same mutations in full-length PIF4 expressed in tobacco. As expected, mKR^ALL^ and mKR^NLS^ markedly diminished mGFPmut2-PIF4 nuclear localization and were therefore excluded from further comparison (Fig. 6g). Both mKR^BH^ and mKR^B^ significantly lowered the number of nuclear speckles but tended to coalesce into larger condensates (Fig. 6g–j). Collectively, these results confirm that basic residues are critical for PIF4’s capacity to phase separate.

### Basic residues in the basic motif and first helix of the PIF4 bHLH domain are indispensable for thermomorphogenesis

**Figure 7.**
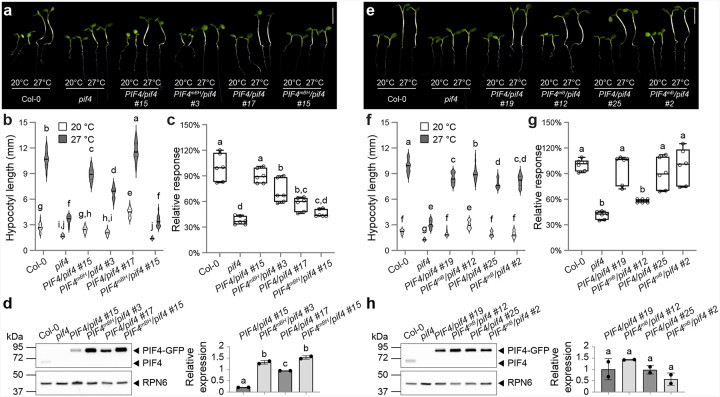
The basic residues in the first helix and the basic motif of the PIF4 bHLH domain are essential for PIF4-mediated thermomorphogenesis. **a**, Representative seedling images. Wild-type (Col-0), *pif4*-*2* (*pif4*), two lines of *PIF4*-*pro::PIF4*-*mGFPmut2*-^*6*^*His/pif4*-*2* (*PIF4/pif4* #15 & #17), and two lines of *PIF4pro::PIF4*^*mBH*^-*mGFPmut2*-^*6*^*His/pif4*-*2* (*PIF4*^*mBH*^*/pif4* #3 & #15) were grown for 4 days in continuous red (Rc) light (50 μmol m^−2^ s^−1^) at 20 °C and 27 °C. **b**, Hypocotyl length measurements of seedlings in **a**. The white and grey violin plots represent hypocotyl length measurements at 20 °C and 27 °C, respectively. The elements of violin plots are as follows: solid line, median; lower dotted line, first quartile; upper dotted line, third quartile. Different letters denote significant statistical differences between the absolute hypocotyl length of each line (two-way ANOVA, n ≥ 33, p < 0.0001). **c**, Comparison of the relative thermal response among the seedlings in **a**. The elements of box plots are as follows: center line, median; box limits, first and third quartiles; whiskers, minimum and maximum values; points, all data points. The relative response is defined as the relative hypocotyl response to 27 °C of a mutant compared with that of Col-0 (which is set at 100%). Different letters denote significant statistical differences between the relative response of each genotype (one-way ANOVA, n = 6, p < 0.05). **d**, Immunoblots showing PIF4 protein levels in transgenic lines in a. Seedlings were grown for 4 days in 50 μmol m^−2^ s^−1^ Rc at 20 °C and treated at 27 °C for 6 hours under the same light conditions. RPN6 was used as an internal control for normalization. The relative PIF4 protein level was calculated based on two immunoblots using two biological replicates. Different letters denote significant statistical differences (one-way ANOVA, n = 2, p < 0.01). **e**, Representative seedling images. Col-0, *pif4*, *PIF4/pif4* #19 & #25, and two lines of *PIF4pro::PIF4*^*mB*^-*mGFPmut2*-^*6*^*His/pif4*-*2* (*PIF4*^*mB*^*/pif4* #12 & #2) were grown for 4 days in Rc (50 μmol m^−2^ s^−1^) at 20 °C and 27 °C. **f**, Hypocotyl length measurements of seedlings in **e**. Different letters denote significant statistical differences between the absolute hypocotyl length of each line (two-way ANOVA, n ≥ 33, p < 0.0001). **g**, Comparison of the relative thermal response among the seedlings in **e**. Different letters denote significant statistical differences between the relative response of each genotype (one-way ANOVA, n = 6, p < 0.01). **h**, Immunoblots showing PIF4 protein levels in transgenic lines in **e**. Seedlings were grown for 4 days in 50 μmol m^−2^ s^−1^ Rc at 20 °C and treated at 27 °C for 6 hours under the same light conditions. RPN6 was used as an internal control for normalization. The relative PIF4 protein level was calculated based on two immunoblots using two biological replicates. One-way ANOVA (n = 2) was performed, and no statistical significance was observed.

Observing that basic residues in the C4 segment are required for PIF4 phase separation, we generated single-insertion *PIF4pro::PIF4*^*mBH*^ and *PIF4pro::PIF4*^*mB*^ lines in the *pif4* background to assess their contribution to thermomorphogenesis (Fig. 7).

We selected two *PIF4pro::PIF4*^*mBH*^ lines (#3 and #15) that accumulated substantially more PIF4 protein than the *PIF4pro::PIF4* control lines (#15 and #17) (Fig. 7d). Despite these high protein levels, both *PIF4pro::PIF4*^*mBH*^ lines displayed hypocotyl lengths similar to the *pif4* mutant at 20 °C and 27 °C (Fig. 7a–c), indicating that the 12 basic residues spanning the basic motif and the first helix of the bHLH domain are essential for PIF4-mediated hypocotyl elongation at warm temperatures. By contrast, two *PIF4pro::PIF4*^*mB*^ lines (#12 and #2), which expressed PIF4 at levels comparable with their wild-type counterparts (#19 and #25), largely rescued the thermomorphogenetic defect: their hypocotyls were as long as—if not longer than—those of the control lines (Fig. 7e–h).

These results yield three insights. First, PIF4’s phase-separation capacity is not strictly required for thermomorphogenesis, because PIF4^mB^–mGFPmut2 remains functional despite its markedly reduced condensate formation (Fig. 6). Second, DNA binding per se might be dispensable, as the basic motif, known for its canonical DNA-binding function in bHLH transcription factors, can be mutated without loss of function in thermomorphogenesis. Third, the failure of PIF4^mBH^–mGFPmut2, but not PIF4^mB^–mGFPmut2, to rescue the *pif4* defects in thermosensory growth points to the three basic residues in the first helix as crucial for PIF4’s thermomorphogenetic activity. This first conclusion is reinforced by our PIF4 TAD-mutant analyses, and we therefore pursued the latter two points in greater detail.

### The DNA-binding capacity of PIF4 is not required for its thermomorphogenetic function

The basic motif of bHLH transcription factors mediates DNA binding^50,73,74^, and several arginine residues in PIF4’s basic region—R254, R257, R269, and R270—are critical for PIF4’s affinity to target promoters^51^. It is therefore striking that replacing nine basic residues in PIF4’s basic motif (variant mB) scarcely affected its thermomorphogenetic activity (Fig. 7e–h).

To probe this further, we drew on work with PIF3, the prototypic PIF protein, where substituting two conserved arginines in the basic region (R353Q/R355Q) abolished DNA binding^75^. These two arginines are highly conserved in the PIF family and align with R267 and R269 in PIF4 (Supplementary Fig. 5c). Because multivalent IDR interactions often scale with length, we reasoned that converting R267 and R269 to glutamines (hereafter RRQQ) would eliminate DNA binding while minimally perturbing phase separation. Consistent with this idea, the RRQQ substitutions, like mB, completely abolished binding of the PIF4 bHLH domain to a G-box (CACGTG) fragment from the *IAA19* promoter in EMSA (Supplementary Fig. 6i). In vitro, the RRQQ version of mGFPmut2-PIF4^C4^ protein formed condensates indistinguishable in density and size from the wild-type protein (Supplementary Fig. 6a–d). When expressed as the full-length PIF4 in tobacco, however, mGFPmut2-PIF4^RRQQ^ produced fewer and larger nuclear speckles, but the effect was milder than mGFPmut2-PIF4^mB^ (Supplementary Fig. 6e–h; Fig. 6g–j).

We analyzed two single-insertion *PIF4pro::PIF4*^*RRQQ*^ lines (#22 and #3), whose PIF4 abundance laid between that of *PIF4pro::PIF4* lines #15 and #17 (Supplementary Fig. 6m). Both *PIF4pro::PIF4*^*RRQQ*^ lines largely rescued the *pif4* thermomorphogenetic defect, exhibiting hypocotyl lengths intermediate between the two controls at 20 °C and 27 °C (Supplementary Fig. 6j-l). Thermo-induced expression of the PIF4 targets *IAA19* and *IAA29* trended lower in *PIF4pro::PIF4*^*RRQQ*^ lines, but the differences were not significant; only *IAA29* expression in control line #17 exceeded that in both *PIF4pro::PIF4*^*RRQQ*^ lines (Supplementary Fig. 6n). Together, these data indicate that PIF4’s DNA-binding activity makes only a minor contribution to thermomorphogenesis.

### The oligomerization ability is central to PIF4-mediated thermomorphogenesis

Our TAD- and basic-region analyses indicate that PIF4 phase separation capacity is largely dispensable for thermosensory hypocotyl growth. By contrast, the divergent behavior of the mBH and mB variants points to three basic residues in the first helix of the bHLH domain—R272, R276, and K278—as critical determinants of PIF4 thermomorphogenetic function. R276 and K278 lie within one of the two dimer–dimer interfaces that assemble the PIF4 bHLH tetramer^51^. Previous work showed that extensive substitution of these interfaces forces the domain to exist only as dimers. We therefore hypothesized that the inability of the mBH variant to rescue the *pif4* mutant stems from a failure to oligomerize.

**Figure 8.**
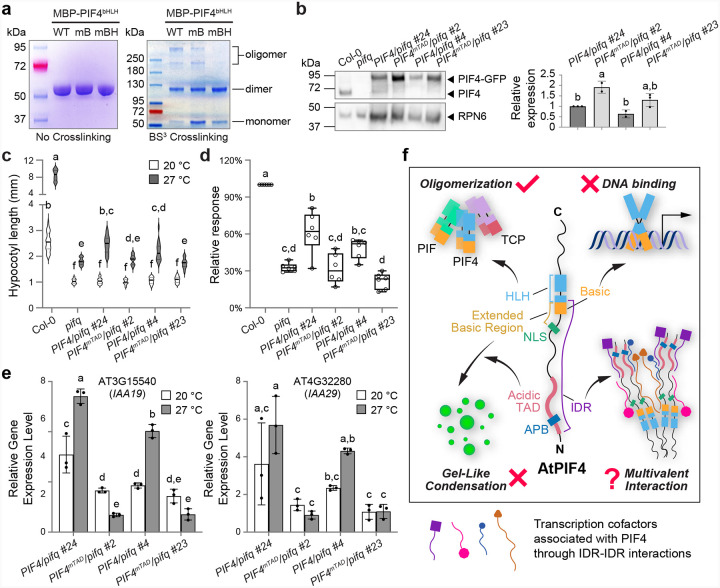
PIF4 oligomerization ability determines its thermomorphogenetic function. **a**, Coomassie blue staining of PAGE gels. The left SDS-PAGE shows that the same amounts of wild-type (WT) and mutant variants (mB and mBH) of MBP-PIF4^bHLH^ proteins were used in the BS^3^ crosslinking assay in the right panel. MBP-PIF4^bHLH^ oligomers are visible in the WT and mB versions, but not in the mBH version. **b**, Immunoblot analysis of PIF4 protein levels in the transgenic lines in **c**. Seedlings were grown at 20 °C and then transferred to 27 °C for 6 hours under Rc. RPN6 was used as a loading control. The relative PIF4 protein level was calculated based on three immunoblots using two and three biological replicates. Different letters denote significant statistical differences (one-way ANOVA, n = 2 or 3, p < 0.05). **c**, Hypocotyl length measurements of seedlings. Seedlings were grown for 4 days in continuous red (Rc) light (50 μmol m^−2^ s^−1^). The white and grey violin plots represent hypocotyl length measurements at 20 °C and 27 °C, respectively. The elements of violin plots are as follows: solid line, median; lower dotted line, first quartile; upper dotted line, third quartile. Different letters denote significant statistical differences between the absolute hypocotyl length of each line (two-way ANOVA, n ≥ 38, p < 0.05). **d**, Comparison of the relative thermal response among the seedlings. The elements of box plots are as follows: center line, median; box limits, first and third quartiles; whiskers, minimum and maximum values; points, all data points. The relative response is defined as the relative hypocotyl response to 27 °C of a mutant compared with that of Col-0 (which is set at 100%). Different letters denote significant statistical differences between the relative response of each genotype (one-way ANOVA, n ≥ 4, p < 0.05). **e**, RT-qPCR analysis of thermo-induced, growth-promoting gene expression levels. Seedlings were grown at 20 °C and then transferred to 27 °C or maintained at 20 °C for 6 hours under Rc. Data was shown from three technical replicates. Different letters denote significant statistical differences (one-way ANOVA, n = 3, p < 0.05). **f**, Proposed sequence features of AtPIF4. The Helix-Loop-Helix (HLH) module mediates dimerization and oligomerization (homotypic with PIFs and heterotypic with TCP). The extended basic region is required and sufficient to drive gel-like condensation, and the acidic TAD regulates the self-enrichment process. The basic motif in the bHLH is responsible for contacting cis-elements in the promoters of target genes. The long N-terminal IDR engages in multivalent interactions with numerous cofactors, such as HSFA1d, MED25, HMR, and MRG2, illustrated as shapes (structured domains) with lines (IDRs). The check mark and crosses indicate that such a function is required or dispensable for PIF4-mediated thermosensory hypocotyl growth, respectively. The question mark indicates that how multivalent interactions through the long IDR contribute to thermomorphogenesis is unclear and requires further investigation. However, the transactivation activity of PIF4 is not essential.

To test this idea, we purified wild-type and mutant variants of MBP–PIF4^bHLH^ and performed a Bis(sulfosuccinimidyl) suberate (BS^3^) crosslinking assay. Like the wild-type MBP–PIF4^bHLH^, the mB version, which alters only the basic motif, showed both dimers and high-molecular-weight multimers, whereas the multimer band was barely visible in the mBH version, which additionally targets the first helix (Fig. 8a). These results confirm that the basic residues in the first helix underpin PIF4 oligomerization and that the loss of oligomerization underlies the functional defect of the mBH variant.

The HLH domain of PIF4 can be used for its hetero-oligomerization with other PIFs^50–52^. We therefore hypothesized that such a partnership might compensate for weakened TAD activity (e.g., mTAD and mWFYL) or DNA-binding (mB and RRQQ) through hetero-oligomerization with PIF4, and that removing close partners would unmask the importance of these functions of PIF4 in thermosensory gene expression. To test this model, we generated single-insertion *PIF4pro::PIF4* and *PIF4pro::PIF4*^*mTAD*^ lines in the *pifq* (*pif1 pif3 pif4 pif5* quadruple) mutant background (Supplementary Fig. 7a). Plotting absolute hypocotyl length at 27 °C against relative *PIF4* transcript abundance (Col-0 = 1) revealed a clear dosage response in *PIF4pro::PIF4/pifq* seedlings, whereas *PIF4pro::PIF4*^*mTAD*^*/pifq* seedlings remained as short as *pifq* irrespective of the *PIF4*^*mTAD*^ transgene level (Supplementary Fig. 7b). We further compared two lines each from *PIF4pro::PIF4*^*mTAD*^*/pifq* (#2 and #23) and *PIF4pro::PIF4/pifq* (#24 and #4). Despite accumulating higher levels of PIF4 protein, the *PIF4pro::PIF4*^*mTAD*^*/pifq* seedlings were drastically shorter than their wild-type counterparts at 27 °C (Fig. 8b–d). Consistently, warm-temperature induction of the PIF4 targets *IAA19* and *IAA29* was abolished in both *PIF4pro::PIF4*^*mTAD*^*/pifq* lines (Fig. 8e). Collectively, these data support the notion that the central role of PIF4 in thermomorphogenesis hinges on HLH-mediated hetero-oligomerization with partner factors (e.g., other PIFs), whereas its other canonical TF features, including transactivation and DNA-binding activities, contribute only marginally to thermosensory growth (Fig. 8f).

## Discussion

Eukaryotic TFs are traditionally defined by two capacities—sequence-specific DNA recognition and cofactor recruitment, both of which are critical for gene expression modulation. Although PIF4, a typical bHLH TF, carries the expected machinery for both tasks—an arginine-rich DNA-binding motif and an acidic TAD—our results show that neither is required for PIF4-mediated thermosensory gene expression or hypocotyl growth. Moreover, these two motifs form part of a long N-terminal IDR that promotes PIF4 phase separation in vitro and *in planta*. Yet, site-directed mutagenesis and genetic complementation reveal that PIF4’s ability to form nuclear condensates is also dispensable for its function in thermomorphogenesis. By contrast, disrupting PIF4 oligomerization by mutating the well-structured HLH domain fully abolishes its thermosensory function. We conclude that PIF4 drives thermomorphogenesis primarily through its ability to assemble higher-order complexes—likely with other PIFs and cofactors—acting as a molecular glue rather than relying on DNA binding, transactivation, or condensate formation (Fig. 8f).

### Contribution of the TAD to PIF4-dependent gene expression at warm temperatures

TADs are responsible for the recruitment of coactivator complexes, chromatin modifiers, and/or basal transcriptional machinery^76^. Owing to their intrinsic disorder and poor sequence conservation, TADs remain difficult to predict *in silico* from primary sequences, even with recent large-scale ED catalogues and deep-learning tools developed in yeast, fly, human, and plant systems^42,68,69,77–80^. Consequently, experimental validation is essential to define their precise boundaries.

Using systematic truncations and alanine scanning (Supplementary Fig. 1; Fig. 2b), we mapped the PIF4 TAD to the first 91 residues, pinpointing two critical segments—TAD1 (22–36 a.a.) and TAD2 (62–91 a.a.) for PIF4’s transcriptional activity in yeast (Supplementary Fig. 1c). Their composition mirrors that of typical acidic TADs, where interleaved acidic and hydrophobic residues are vital for activity, as described in the “Acidic Exposure” model^68,69^. According to this model, critical aromatic and leucine residues dock into shallow hydrophobic grooves on coactivators, while neighboring acidic residues repel each other and keep these hydrophobic residues solvent-exposed^68,70^. In line with this model, alanine replacement of multiple aromatic, leucine, and/or acidic residues (e.g., the m7 WRDGQ-AAAAA and m14 EDQET-AAAAA variants) strongly impaired PIF4-mediated transcription in yeast (Supplementary Fig. 1c). Conversely, other substitutions had little or even opposite effects; for example, the m4 variant (NRRSI-AAAAA) significantly enhanced PIF4 activity in yeast (Supplementary Fig. 1c). Besides, a more extreme mutation underscores the regulatory role of the acidic patch: substituting ten acidic residues to their amide counterparts (D to N, E to Q) in PIF4 TAD generated the mDE variant (Fig. 4b), which displayed normal mRNA levels in *Arabidopsis* yet accumulated little protein (Fig. 5d,e). Removing so many acidic residues likely allows hydrophobic patches to collapse both intramolecularly and intermolecularly, promoting aggregation and subsequent degradation by the cellular quality-control machinery. Our observations of the mGFPmut2-PIF4^IDR^ recombinant proteins in bacteria support this notion: the mDE version was less stable compared with the wild-type and the mWFYL variant (Fig. 4d). Thus, the acidic residues in the PIF4 TAD not only license coactivator engagement but also safeguard local conformation by preventing inappropriate exposure of hydrophobic residues.

The PIF4 TAD is also rich in serine, proline, and asparagine residues (Fig. 4b), hinting at overlapping features of serine-rich, proline-rich, and Q-rich TADs^76^. However, alanine scanning provided little support for a critical role of these residues; for instance, m10 (QTQTQ-AAAAA) had no impact on PIF4 activity in yeast (Supplementary Fig. 1c). We cannot fully exclude context-specific functions, especially because serines may be post-translationally modified (e.g., phosphorylation and glycosylation) and potentially affect PIF4’s transcriptional activity in *Arabidopsis*.

Given the importance of acidic and hydrophobic residues in PIF4 TAD, we expected that their alanine replacement would cripple PIF4-mediated thermomorphogenesis. To our surprise, neither the mTAD nor the hydrophobic-cluster mutant mWFYL compromised PIF4 function in *Arabidopsis*; both largely restored thermosensory hypocotyl growth in *pif4* (Fig. 3j–n; Fig. 5g–k). Because PIF4 hetero-oligomerizes with other PIFs, and because disrupting its oligomerization capacity severely curtailed its thermomorphogenetic function in *Arabidopsis* (Fig. 7a–d; Fig. 8a), we propose that the reduced TAD function of PIF4 would be compensated by other PIFs’ TADs in *Arabidopsis*, minimizing the negative impact on plant thermomorphogenesis. Supporting this view, PIF4^mTAD^ failed to activate thermo-induced gene expression or hypocotyl elongation in the *pifq* background, where its closest partners (PIF1, PIF3, and PIF5) are also absent (Fig. 8b–e).

### Contribution of PIF4’s DBD to its role in thermosensory gene expression

Most DBDs, including the basic motif in bHLH, carry a net positive charge under physiological conditions, allowing their basic side chains to contact the acidic sugar-phosphate backbone of DNA nonspecifically^81^. Classic studies on the bHLH proteins myoD and E47 demonstrated that neutralizing key basic residues abolishes DNA-binding^73,82^, and the same is true for PIF3, where the double substitution, R353Q and R355Q, eliminates DNA-binding affinity^75^. We introduced the corresponding mutations in PIF4, creating the RRQQ variant, and also generated a basic-cluster mutant (mB); both lost DNA-binding capacity in vitro (Supplementary Fig. 6i). Remarkably, each mutant retained thermomorphogenetic function, largely, if not fully, restoring thermosensory hypocotyl growth in *pif4* (Fig. 7e–h; Supplementary Fig. 6j–n). These observations suggest that specific DNA recognition by PIF4 per se is not essential for its role in thermo-induced gene activation in *Arabidopsis*. This outcome also contrasts sharply with that of atypical bHLH, or HLH, proteins, which naturally lack the basic motif. HLH proteins, such as PHYTOCHROME RAPIDLY REGULATED 1 (PAR1), heterodimerize with other bHLH proteins (e.g., PIF4) and antagonize their transcriptional activity^83,84^. By contrast, the DNA-binding-deficient mB variant, despite retaining the HLH dimerization module, did not antagonize other PIFs’ functions; instead, it fully complemented the *pif4* phenotype. Because both RRQQ and mB preserve an intact HLH interface (Fig. 8a), we infer that their loss of DNA binding is masked by partner PIFs that supply this activity in the hetero-oligomeric complex.

Such functional buffering effects appear common. The DNA-binding-deficient PIF3 mutant mentioned above could also fully rescue *pif3* hypocotyl defects in darkness and red light^75^. Likewise, the leukemia oncoprotein SCL, a vertebrate bHLH TF, complements hematopoietic and vascular deficits in zebrafish despite abolished DNA binding^85^. SCL forms a pentameric assembly with two closely related bHLH TFs (E12 and LMO2) and two other TFs (Ldb1 and GATA-1), echoing the PIF4 paradigm. Therefore, the PIF4 mode of thermosensory gene regulation—where redundant DBDs buffer the transcriptional complex against loss of DNA association in any single component—might be prevalent among bHLH TF families across kingdoms. Conversely, our data argue against PIF4 acting as a pioneer TF—defined by sequence-specific, high-affinity DNA-binding ability^39^.

### Contribution of oligomerization to PIF4’s role in thermomorphogenesis

Although certain TFs can bind DNA as monomers, most engage their targets as dimers or higher-order multimers^47^. Dimerization stabilizes DNA contacts, broadens sequence recognition through partner exchange, and can trigger cofactor recruitment^86^—for example, activating the p300 acetyltransferase^87^.

Higher-order oligomerization confers even greater advantages: it promotes cooperative and more specific DNA binding, eases coregulator recruitment, and helps organize DNA looping and chromatin remodeling^88–92^. Our results add a further benefit—functional buffering. In the HLH-mediated PIF4 oligomers, loss of transactivation or DNA-binding activity in one subunit can be masked by the intact domains of its partners. Consequently, mutations limited to the PIF4 TAD or basic motif do not abolish thermomorphogenesis (Fig. 3j–n; Fig. 5g–k; Fig. 7e–h; Supplementary Fig. 6j–n), because the oligomer retains at least one functional TAD and DBD. This does not mean that PIF4’s own TAD or DBD is irrelevant; rather, within the multivalent complex, each module contributes, but no single element is indispensable, allowing the assembly to remain operative even when one component is compromised.

The buffering model might suggest that PIF4’s heterooligomeric partners contribute on par with PIF4 to thermosensory gene expression, especially because other PIFs share similar HLH oligomerization domains. Yet genetics highlight PIF4’s privileged role—within the PIF quartet (PIF1, PIF3, PIF4, and PIF5), only loss of *PIF4* markedly dampens thermosensory hypocotyl elongation, whereas single knockouts of the others have minimal effect^7,93^. These observations point to PIF4-specific features that determine its central role in thermomorphogenesis. First, PIF4 protein abundance is strongly induced by warm temperatures^93^. Second, its HLH might possess unique characteristics that support heterotypic oligomerization with non-PIF cofactors. Third, its long N-terminal IDR could broaden multivalent interaction capacity. Consistent with these ideas, PIF4 interacts with numerous TTFs^7,8,12–17^, co-activators^93–95^, Mediator components^55,56^, and histone modifiers^96^. We will discuss this further in the last section.

### Gel-like PIF4 condensates and their role in thermal responses

Liquid-liquid phase separation (LLPS) compartmentalizes biomacromolecules into membraneless organelles^97^. In animals and fungi, many TFs undergo LLPS with cofactors and chromatin to create dynamic transcriptional hubs that modulate gene expression^98^. Similarly, *Arabidopsis* HSFA1a forms liquid condensates that boost gene expression in response to heat stress, potentially by enabling the formation of DNA loops between heat-responsive promoters and enhancer motifs^60^. By contrast, PIF4 assembles into gel-like spheres with very low internal mobility, as shown by FRAP, and their liquidity remains unchanged across the warm-temperature range, suggesting a fast rigidification process—almost instantaneous liquid-to-solid transition (Fig. 1).

Condensate material properties can be modulated by binding partners. Coexpression of JAZ3, a jasmonic acid signaling component, liquefies PIF4 droplets, yet JAZ3 simultaneously suppresses PIF4’s transcriptional activation and DNA binding activities, acting as an attenuator of PIF4-mediated transcriptional regulation^58^. The same phenomenon has been observed in ARF19 and ELF3 condensates, both of which sequester TFs from accessing transcriptional targets^24,99^. Although the JAZ3 study claimed PIF4 could not phase separate on its own^58^, our in vitro and *in planta* imaging clearly show autonomous PIF4 condensate formation (Fig. 1). Technical hurdles—most notably the weak fluorescent signal of PIF4-mGFPmut2 in transgenic plants—prevented us from monitoring PIF4 condensate dynamics in *Arabidopsis*. However, we sought to understand whether the PIF4 condensate formation is really important for its transcriptional activity by performing mutagenesis and functional complementation. We were able to identify two critical sequence features that are crucial to PIF4 condensate formation in vitro and in tobacco nuclei. Disrupting either the acidic TAD (mWFYL) or the basic DNA-binding motif (mB) significantly abolishes PIF4 droplet formation (Fig. 4 and Fig. 6), yet each mutant variant could still complement the *pif4* thermomorphogenetic defect (Fig. 5 and Fig. 7). Phase separation propensity is therefore not essential for PIF4-mediated thermosensory growth.

### Condensate-independent roles of the PIF4 N-terminal IDR in thermomorphogenesis

Phase separation is often attributed to multivalent interactions among IDRs, although some globular proteins lacking extended disorder can also undergo LLPS^100^. PIF4 carries a long N-terminal IDR that alone self-enriches in gel-like condensates, recapitulating the behavior of the full-length protein (Fig. 1). This contrasts with findings in *Drosophila* embryos, where isolated IDRs are insufficient to drive subnuclear clustering^101^. Although our data indicate that condensate formation per se is dispensable for PIF4-driven thermomorphogenesis, they do not exclude a functional role for IDR-mediated multivalent contacts. Some TFs stimulate transcription through such interactions even in the absence of biomolecular condensates^64^. Therefore, it is possible that PIF4’s IDR still contributes to its thermomorphogenetic function, not by condensate formation, but via multivalent interactions (Fig. 8f).

Our functional buffering model implies that PIF4 harbors unique sequence and/or structural determinants that enable extensive cofactor engagement during thermal responses. Are these features embedded in the HLH dimerization/oligomerization domain, the IDR, or both? While we did not exhaustively dissect this question, promoter- and domain-swapping experiments between PIF1 and PIF4, performed by Giltsu Choi’s group, offered some clues^102^. The promoter swapping assay demonstrated that both the *PIF4* promoter and PIF4 protein were required to confer its dominant role in thermosensory hypocotyl growth. Moreover, domain swapping showed that the N-terminal region upstream of bHLH conferred functional characteristics to PIF4, as the chimeric protein having PIF4 N-terminus fused to PIF1 C-terminus rescued *pif1 pif4* defects in thermomorphogenesis, whereas the reciprocal fusion did not^102^. These data implicate the PIF4 N-terminal IDR in recruiting cofactors for promoting thermomorphogenesis, while the HLH module is necessary but only needed for its dimerization/oligomerization function.

At first glance, our TAD and DBD mutation results seem at odds with this view because individual lesions in either region only had a mild impact on PIF4’s thermomorphogenetic function (Fig. 5 and Fig. 7). However, we perturbed only the extreme N- and C-terminal edges of the long IDR; more than 100 residues in between remain untested. Because IDR interaction strength often scales with length, partial disruption—via mutations in the N-terminal TAD or the C-terminal basic motif—may leave sufficient interaction potential intact, explaining the marginal phenotypic impact of these mutations. Determining whether and how the intact IDR assembles a condensate-independent network of transient contacts that underpins PIF4’s central role in thermomorphogenesis remains an important goal for future work.

## Methods

### Plant materials and growth conditions

The *Arabidopsis* mutants *pif4*-*2* (SAIL_1288_E07) and *pifq* have been previously described^93^. Homozygosity was confirmed by PCR before the seeds were used for phenotyping and *Agrobacterium*-mediated floral dipping. Seeds were surface sterilized with bleach (3% sodium hypochlorite) for 8 min before being plated on half-strength sucrose-free Murashige and Skoog (1/2 MS0) media supplemented with Gamborg’s vitamins (Caisson Laboratories, North Logan, UT), 0.5 mM MES (pH 5.7), and 0.8% (w/v) agar (Caisson Laboratories, North Logan, UT). Seeds were stratified in the dark at 4 °C for 3–5 days to synchronize germination before treatment under specific light and temperature conditions in LED chambers (Percival Scientific, Perry, IA). Fluence rates of light were measured using an Apogee PS200 spectroradiometer (Apogee Instruments Inc., Logan, UT).

### Hypocotyl measurements

Seeds were plated on the surface of ½ MS0 media and grown at either 20 °C or 27 °C under continuous red light (50 μmol m^−2^ s^−1^) for 96 hours. More than fifteen seedlings from each line were placed on transparent film and scanned using an Epson Perfection V700 photo scanner. Hypocotyl length was measured using NIH ImageJ software (http://rsb.info.nih.gov/nih-image/). The percent increase (PI) in hypocotyl length was calculated as the percentage change observed at 27 °C relative to the corresponding measurement at 20 °C. The relative response of each mutant was defined as the percentage of its PI value normalized to that of the Col-0 wild type. At least three replicates were used to calculate the mean and standard deviation of each relative response. Violin and box plots were generated using Prism 10 (GraphPad Software, San Diego, CA).

### Plasmid constructions

All the primers used for cloning are listed in the Supplementary Table 1. PCR amplifications were performed using the Q5 High-Fidelity DNA Polymerase and the ligation reactions with the NEBuilder HiFi DNA Assembly Master Mix (New England Biolabs Inc., Ipswich, MA). To generate various mGFPmut2-PIF4 recombinant proteins, the coding sequence (CDS) of *mGFPmut2*, the *GS*-*linker* (GAPGSAGSAAGGSG), and the wild-type or mutant *PIF4* were cloned into pET28a vectors between EcoRI and XhoI. To generate MBP-PIF4^bHLH^ recombinant proteins, the wild-type or mutant coding sequence of the *PIF4* bHLH domain was amplified and cloned into the pMAL-C2X vector between EcoRI and BamHI. For transient protein expression and luciferase assay, the sequence of *mGFPmut2*-*GS*-*PIF4* was cloned into pCHF1 or pCHF3 between BamHI and SalI. For the luciferase assay, the luciferase gene and an *RbcS* terminator sequence were cloned into pJHA212B between PstI and HindIII, and a 2-kb *IAA19* promoter was added between EcoRI and SalI. Mutations or fragments of *PIF4* CDS were cloned into pBridge between EcoRI and SalI for alanine scanning and transactivation assay. To generate transgenic lines, a 2-kb promoter region of *PIF4* was fused with *PIF4* CDS (wild-type and mutant versions) and cloned into pJHA212H between EcoRI and BamHI along with a C-terminal *(PT)*_*4*_*P linker*, *mGFPmut2*, and a *His*-*tag*.

### Recombinant protein expression and purification

For recombinant protein expression, the aforementioned pET28a or pMAL-C2X plasmids were transformed into BL21 (DE3) Rosetta strains of *E. coli* by electroporation. The expression of recombinant proteins cloned into pET28a was induced at 37 °C for 3 h with 0.5 mM IPTG. Cells were harvested and lysed in lysis buffer (50 mM Tris-HCl, pH 7.5, 300 mM NaCl, 10 mM imidazole, and 2 mM PMSF) using a Pulse 150 Ultrasonic Homogenizer with Φ6 probe (Benchmark Scientific, Sayreville, NJ). The supernatant was harvested and incubated with lysis buffer-equilibrated Ni-NTA His-Pur agarose (ThermoFisher Scientific, Waltham, MA) for 1 h at 4 °C. The protein-agarose slurry was packed in a gravity-flow column (Bio-Rad Laboratories, Hercules, CA) and washed with 3 volumes of wash buffer (50 mM Tris-HCl, pH 7.5, 300 mM NaCl, and 25 mM imidazole) and eluted using elution buffers with increasing imidazole concentrations (50 mM Tris-HCl, pH 7.5, 300 mM NaCl, 50–250 mM imidazole, and 1 mM DTT). Eluates were combined based on SDS-PAGE results, immediately concentrated and desalted in a storage buffer (50 mM Tris-HCl, 125 mM NaCl, 10% glycerol, and 1 mM DTT) using 30K Amicon Ultra centrifugal filters (Sigma Aldrich Inc., St. Louis, MO), flash-frozen in liquid nitrogen, and stored at −80 °C until use. Protein concentrations were measured using the Bradford assay and verified by SDS-PAGE.

For proteins cloned into pMAL-C2X, the initial purification followed the same Ni-NTA His-tag protocol as described above. After the eluates were desalted, MBP-tagged proteins were further purified using amylose resin according to the manufacturer’s instructions (New England Biolabs Inc., Ipswich, MA). Briefly, the protein-resin was packed into a gravity-flow column, washed with wash buffer (20 mM Tris-HCl, pH 7.5, 150 mM NaCl), and the protein was eluted with the same wash buffer containing 10 mM maltose. Final eluates were pooled, and protein quality was verified by SDS-PAGE after being quantified using the Bradford assay.

### In vitro droplet formation

Purified protein samples were diluted to the required concentrations and mixed with the respective volume of droplet formation buffer (50 mM Tris-HCl, pH 7.5, 125 mM NaCl, 10% glycerol, 10% PEG-8000, and 1 mM DTT). Imaging was performed in a glass-bottom 384-well plate (Cellvis, Mountain View, CA) or on a glass slide (Fisherbrand, Pittsburgh, PA) using a Leica SP8 confocal microscope (Leica Microsystems Inc., Buffalo Grove, IL). All the images were taken at the bottom-most plane (on the surface of the 384-well plate or slide). Excitation and emission wavelengths for mGFPmut2 were 488 nm and 500–550 nm, respectively.

### In planta condensate formation

Binary constructs based on the pCHF1 vector carrying *PIF4* coding sequences or its mutant variants were transformed into the GV3101 *Agrobacterium* strain by electroporation and screened on LB media containing 20 μg/mL gentamycin, 25 μg/mL rifampicin, and 100 μg/mL spectinomycin. Individual colonies were inoculated to grow a small-scale culture, which was harvested at an OD_600_ of 0.6, resuspended in infiltration buffer (10 mM MES, pH 5.7, 10 mM MgCl_2_), and treated with 150 μM acetosyringone for 2–4 h. The suspension was injected into tobacco leaves and incubated at 20 °C for 36–48 h. For temperature treatments, tobacco plants were first incubated at 20 °C for 24 h, and then transferred to 28 °C for another 12 h. A portion of the leaf near the injection site was cut, mounted in water, and imaged using the 40× immersion lens on the Leica SP8 confocal microscope. Excitation and emission wavelengths for mGFPmut2 were 488 nm and 500–550 nm, respectively.

### Fluorescent Recovery After Photobleaching (FRAP) assays

FRAP experiments were performed using the Leica SP8 confocal microscope with a 40× water-immersion objective. The GFP signal was bleached using a 488 nm laser (100% laser power). For in vitro samples, bleaching was performed with 2–4 iterations, while for tobacco samples, 8–10 iterations were used to ensure sufficient bleaching. Fluorescence recovery was recorded every 10 seconds for 20 time points. Recovery curves were generated using the ImageJ software.

### Luciferase assay in Nicotiana benthamiana

For the luciferase assay, *Agrobacterium* strains carrying pCHF1/PIF4-(PT)_4_P-mGFPmut2-^6^His and pJHA212B/IAA19pro::LUC-RbcSter constructs were mixed and co-infiltrated into tobacco leaves, using the empty pCHF1 and pJHA212B/IAA19pro::LUC-RbcSter as a control. After 2 days of incubation, the infiltrated leaves were cut and sprayed with 3 mM luciferin substrate. Luminescence signals were detected using an Azure C600 Advanced Imaging System (Azure Biosystems, Dublin, CA, USA), and the signal strength was quantified using the ImageJ software.

### Yeast Transactivation Assay

Yeast transactivation and alanine scanning assays were conducted using either Y2HGold yeast strains (TaKaRa Bio USA, San Jose, CA) harboring a pBridge bait vector or diploid yeast strains obtained by mating Y2HGold (bait) with Y187 (prey) strains, the latter carrying the pGADT7 vector (TaKaRa Bio USA, San Jose, CA). Overnight cultures were adjusted to an OD_600_ of 0.2 and subjected to tenfold serial dilutions. A 10-μL aliquot from each dilution was spotted onto selective synthetic dropout media—Y2HGold onto SD/-Trp media, and diploid strains onto SD−/Trp/−Leu (± 250 ng/mL Aureobasidin A) and SD/−Trp/−Leu/−His (+ 10 mM 3-Amino-1,2,4-triazole) media. Plates were incubated at 30 °C, and colony growth was documented on the third day.

For liquid β-galactosidase assays, diploid cells were generated by mating Y2HGold and Y187 strains and selected on SD/−Trp/−Leu plates. These cells were then grown overnight in liquid SD/−Trp/−Leu medium, and β-galactosidase activity was quantified using ortho-nitrophenyl-β-galactoside (ONPG) as the substrate, following the Yeast Protocols Handbook (TaKaRa Bio USA, San Jose, CA).

### Generation of transgenic lines

To generate wild-type and mutant versions of *PIF4pro::PIF4*-*mGFPmut2* transgenic lines, the *pif4*-*2* and *pifq* mutant plants were transformed with wild-type or mutant versions of *pJHA212H/PIF4pro::PIF4*-*(PT)*_*4*_*P*-*mGFPmut2*-^*6*^*His* plasmids using the *Agrobacterium*-mediated floral dip method. Positive transformants were selected on the ½ MS0 medium containing 50 μg/mL hygromycin. At least six independent lines that segregated approximately 3:1 for hygromycin-resistance in the T2 generation were identified, and at least two lines were selected for this study based on the transgene expression level. For all experiments, T3 self-progenies of homozygous T2 plants were used.

### RNA extraction and quantitative reverse transcription PCR

50–100 mg of seedlings were used to extract total RNA using the Quick-RNA Miniprep Kit (Zymo Research, Irvine, CA), with on-column DNase I treatment. First-strand cDNA synthesis was carried out using 0.5–2.5 μg of total RNA using the SuperScript III Reverse Transcriptase and the Oligo(dT)_20_ primer (Thermo Fisher Scientific, Waltham, MA). For qPCR, diluted cDNA was mixed with 2× Universal SYBR Green Fast qPCR Mix (ABclonal Technology, Woburn, MA) along with gene-specific primers listed in Supplementary Table 2. Triplicates of each RT-qPCR reaction were performed using the Quant Studio 3 (Thermo Fisher Scientific, Waltham, MA). Gene expression was normalized to *PP2A* transcript levels and quantified using the 2^−ΔΔCt^ method. Final data were visualized as bar graphs using Prism 10 software (GraphPad Software, San Diego, CA).

### Protein extraction and immunoblotting

For immunoblot analyses, total protein was extracted from 4-day-old seedlings as previously described^55^. In brief, 200 mg of fresh seedlings were flash frozen and ground to a fine powder using sterile wooden pestles and vortexed in three volumes of extraction buffer containing 100 mM Tris-HCl (pH 7.5), 100 mM NaCl, 5 mM EDTA, 5% SDS, 20% glycerol, 20 mM DTT, 40 mM β-mercaptoethanol, 2 mM phenylmethylsulfonyl fluoride, 40 μM MG115 (Apexbio Technology LLC, Houston, TX), 40 μM MG132 (Cayman Chemical, Ann Arbor, MI), 1× phosphatase inhibitor cocktail 2 (Thermo Fisher Scientific), 1× phosphatase inhibitor cocktail 3 (MilliporeSigma), 1× EDTA-free protease inhibitor cocktail (MilliporeSigma), and 0.01% bromophenol blue. Protein extracts were then boiled for 10 minutes and clarified by centrifugation at 20,000 × g for 10 minutes.

Cleared protein samples were separated via SDS-PAGE, transferred to nitrocellulose membranes at 70V for 1–2 h, probed with the indicated primary antibodies overnight, and then incubated with a 1:5000 dilution of horseradish peroxidase-conjugated goat anti-rabbit secondary antibodies (Bio-Rad Laboratories, 1706515). Primary antibodies, including polyclonal rabbit anti-PIF4 antibodies (Agrisera, AS163157 and Abiocode, R2534–1) and polyclonal rabbit anti-RPN6 antibodies (Enzo Life Sciences, BMLPW8370–0100), were used at 1:1000 dilutions. Signals were detected via chemiluminescence using a SuperSignal kit (Thermo Fisher Scientific, Waltham, MA) and an Azure C600 Advanced Imaging System (Azure Biosystems, Dublin, CA).

### Electrophoretic mobility shift assay (EMSA)

5′-biotin-labeled complementary oligonucleotides corresponding to the G-box-containing region of the *IAA19* promoter were synthesized (Sigma-Aldrich, St. Louis, MO, USA). The wild-type probe sequence was 5′–tatttcatataatttCACGTGgcccaacttgtttct–3′, and the mutated probe carried a disrupted G-box motif: 5′–tatttcatataatttACAACAgcccaacttgtttct–3′. To generate double-stranded DNA probes, oligonucleotides were annealed by heating at 95 °C for 5 min, followed by slow cooling to room temperature. EMSAs were performed using the LightShift Chemiluminescent EMSA kit (Thermo Fisher Scientific, Waltham, MA). Briefly, binding reactions (20 μL total volume) were carried out in binding buffer (2 μL 10× binding buffer, 5 mM MgCl_2_, 1 mM EDTA, 0.05% NP-40, 5% glycerol, 50 ng poly(dI-dC)), 1–2 μg purified protein, and 20 fmol of biotin-labeled probe. The reaction mixture was incubated at 4 °C for 20–30 min. Binding reactions were loaded on a 6% native polyacrylamide gel and subsequently transferred to a nylon membrane (Merck Millipore, Burlington, MA). Signal was detected using an Azure C600 Advanced Imaging System (Azure Biosystems, Dublin, CA).

### BS^3^ crosslinking assay

Purified protein samples were buffer exchanged into a crosslinking-compatible buffer (20 mM HEPES, pH 7.5, 150 mM NaCl) using 30K Amicon Ultra centrifugal filters (Sigma Aldrich Inc., St. Louis, MO). Bis(sulfosuccinimidyl) suberate sodium salt (BS^3^) (Thermo Fisher Scientific, Waltham, MA) was freshly prepared as a 25 mM stock solution in PBS immediately before use. For crosslinking, the protein was mixed with 0.25 mM BS^3^ and incubated at 4 °C for 2 h. The reaction was quenched by adding 1 M Tris-HCl (pH 7.5) and further incubated at room temperature for 15 minutes. Samples were mixed with 4× non-reducing SDS loading buffer (250 mM Tris-HCl, pH 6.8, 10% SDS, 40% Glycerol), subjected to SDS-PAGE, and visualized with Coomassie blue staining.

## Supplementary Material

Supplementary Files

This is a list of supplementary files associated with this preprint. Click to download.

- TableS1.pdf
- TableS2.pdf
- SupplementaryFigures.pdf

**Supplementary Fig. 1 Identification of the residues necessary for PIF4’s TAD activity via alanine-scanning mutagenesis. a**, The N-terminal 78 amino acids of PIF4 confer strong transactivation activity in yeast. **b**, Further fragmentations of the N-terminal 78 amino acids completely abolish the transactivation activity in yeast. **c**, Three yeast reporters, Aureobasidin A (AbA) resistance, HIS3 (-His), and LacZ (β-galactosidase liquid assay), are used to determine the activity of PIF4 wild-type and mutant versions. **d**, Mutating individual hydrophobic residues in M15 does not affect the transactivation activity of PIF4 in yeast.

**Supplementary Fig. 2 Characterizations of *PIF4* transgenic lines in the *pif4* mutant background. a**, Hypocotyl length measurements of seedlings. Wild-type (Col-0), *pif4*-*2* mutant, and transgenic lines expressing *PIF4*-*mGFPmut2*-^*6*^*His* or *PIF4*^*mTAD*^-*mGFPmut2*-^*6*^*His* driven by the *PIF4* native promoter in the *pif4*-*2* mutant background were grown for 5 days in continuous red (Rc) light (50 μmol m^−2^ s^−1^). The white and grey boxes represent hypocotyl length at 20 °C and 27 °C, respectively. The elements of box plots are as follows: center line, median; box limits, first and third quartiles; whiskers, minimum and maximum values; points, all data points. **b**, Relationship between *PIF4* transcript level and hypocotyl length at warm temperatures. The hypocotyl length of each genotype grown at 27 °C in **a** was plotted against the relative *PIF4* transcript level measured in the same condition (that in Col-0 was set to 1). Nonlinear regression (sigmoidal, 4PL) was performed separately for *PIF4*-*mGFPmut2*-^*6*^*His/pif4* (blue) and *PIF4*^*mTAD*^-*mGFPmut2*-^*6*^*His*/*pif4* lines (yellow). Arrows and numbers indicate the transgenic lines selected for further studies. **c**, Hypocotyl length measurements of seedlings grown for 4 days in white light (16-h light/8-h night, 125 μmol m^−2^ s^−1^). The white and grey violin plots represent hypocotyl length measurements at 20 °C and 27 °C, respectively. The elements of violin plots are as follows: solid line, median; lower dotted line, first quartile; upper dotted line, third quartile. Different letters denote significant statistical differences between the absolute hypocotyl length of each line (two-way ANOVA, n ≥ 38, p < 0.01). **d**, Comparison of the relative thermal response among the seedlings. The elements of box plots are as follows: center line, median; box limits, first and third quartiles; whiskers, minimum and maximum values; points, all data points. The relative response is defined as the relative hypocotyl response to 27 °C of a mutant compared with that of Col-0 (which is set at 100%). Different letters denote significant statistical differences between the relative response of each genotype (one-way ANOVA, n = 4, p < 0.05).

**Supplementary Fig. 3 A conserved acidic TAD can be identified in PIF4 homologs from different plant species**. Schematic representation of a computational analysis dividing PIF4 from various plant species into overlapping 39-amino-acid (a.a.) tiles, spaced by 1 a.a. All tiles were analyzed for hydrophobic (W, F, L) and acidic (D, E) residue content. Regions meeting the criteria of an acidic transactivation domain, as predicted by Kotha and Staller (2023), are shaded red and blue. Criteria include W+F+L ≥ 6 and −13 ≤ Charge ≤ −8.

**Supplementary Fig. 4 Determining the minimal region of PIF4 IDR required for phase separation. a**, Schematic representation of truncation constructs derived from the intrinsically disordered region (IDR) of *Arabidopsis thaliana* PIF4 (AtPIF4). Abbreviations: APB, active phyB-binding motif; NLS, nuclear localization signal; bHLH, basic helix-loop-helix domain. Each truncation was fused to mGFPmut2 at the N-terminus to generate recombinant proteins for phase separation analysis. **b**, Coomassie blue staining of purified recombinant proteins used in phase separation assays. **c**, In vitro phase separation assays of mGFPmut2-PIF4^IDR^ truncation proteins. Recombinant proteins (10 μM) were incubated in 10% PEG-8000 with 125 mM NaCl.

**Supplementary Fig. 5 Analysis of amino acid composition in PIF4 basic region. a**, Amino acid composition in the C4 fragment of AtPIF4. Arginine and lysine residues are highlighted in orange. **b**, Heatmap showing the amino acid composition in the C4 regions of PIF4 homologs. Species include *Arabidopsis thaliana* (NP_001323426.1), *Camelina sativa* (XP_010506153.1), *Brassica rapa* (XP_018513637.1), *Nicotiana tabacum* (XP_016471316.1), *Solanum lycopersicum* (XP_019070178.1), *Vitis riparia* (XP_034702447.1), *Populus alba* (XP_034915052.1), *Oryza sativa* (XP_015618074.1), *Brachypodium distachyon* (KQK14025.1), and *Physcomitrella patens* (Pp3c1_38820V3.1.p). **c**, Sequence alignment of eight *Arabidopsis* PIF proteins (AtPIL1 is also known as AtPIF2) in the basic region. Red arrows indicate two arginine residues substituted with asparagines in Fig. 8.

**Supplementary Fig. 6 The DNA-binding ability of PIF4 is dispensable for its thermomorphogenetic function. a**, In vitro phase separation assay of the wild-type and RRQQ mutant versions of mGFPmut2-PIF4^C4^ recombinant proteins. Proteins (20 μM) were incubated in 10% PEG-8000 with 125 mM NaCl. The Coomassie blue staining inside **b** shows protein quality. **b**, Size distribution of mGFPmut2-PIF4^C4^ granules. Wild-type and RRQQ mutant granules were binned into 12 size categories, with the mean percentage for each size bin represented by black (wild-type) or orange (RRQQ) lines, and the standard deviation (SD) shown as shaded areas. Statistical analysis was performed using two-way ANOVA (n = 10; ****, p < 0.0001). **c**, Comparison of granule density between wild-type and RRQQ versions of mGFPmut2-PIF4^C4^. **d**, Average granule sizes of wild-type and RRQQ versions of mGFPmut2-PIF4^C4^. Statistical significance was assessed using a Student’s t-test (n = 10; *, p < 0.05; n.s., not significant). **e**, Confocal microscopy images of wild-type and RRQQ versions of mGFPmut2-PIF4 speckles in *N. benthamiana*. **f–h**, Quantitative analysis of mGFPmut2-PIF4 speckles in *N. benthamiana*. **f**, Proportions of smaller (≤ 0.03 μm^2^) and larger (> 0.03 μm^2^) speckles. **g**, Number of granules per nucleus. **h**, Average granule size. **i**, EMSA showing MBP-tagged PIF4 bHLH domain binds to G-box of the *IAA19* promoter (WT probe, 5’tatttcatataatttCACGTGgcccaacttgtttct), but not the mutated *IAA19* promoter (MT probe, tatttcatataatttACAACAgcccaacttgtttct). MBP, MBP-bHLH^RRQQ^, and MBP-bHLH^mB^ all fail to bind to the WT probe. **j**, Representative images of 4-day-old seedlings grown under continuous red light (Rc, 50 μmol m^−2^ s^−1^) at 20 °C and 27 °C. Genotypes include wild-type (Col-0), *pif4*-*2* (*pif4*), two lines expressing *PIF4pro::PIF4*-*mGFPmut2*-^*6*^*His* in the *pif4*-*2* background (*PIF4/pif4* #15 & #17), and two lines expressing *PIF4pro::PIF4*^*RRQQ*^-*mGFPmut2*-^*6*^*His* in the pif4-2 background (*PIF4*^*RRQQ*^*/pif4* #22 & #3). **k**, Hypocotyl length measurements of seedlings shown in **j**. White and gray violin plots represent data from 20 °C and 27 °C, respectively. Solid lines denote the median, and dotted lines indicate the first and third quartiles. Different letters indicate statistically significant differences in absolute hypocotyl lengths (two-way ANOVA, n ≥ 65, p < 0.05). **l**, Relative thermal response of hypocotyl elongation among the genotypes in **j**. Box plots show the median (center line), first and third quartiles (box limits), and whiskers (minimum and maximum values). Relative response is calculated as the ratio of hypocotyl elongation at 27 °C to 20 °C, normalized to Col-0 (set to 100%). Different letters indicate significant differences between genotypes (one-way ANOVA, n = 3, p < 0.001). **m**, Immunoblot analysis of PIF4 protein levels in seedlings in **j**. Seedlings were grown at 20 °C and then transferred to 27 °C for 6 hours under Rc. RPN6 was used as a loading control, and relative PIF4 levels were quantified from four biological replicates. Statistical differences were determined by one-way ANOVA (n = 4, p < 0.05). **n**, RT-qPCR analysis of thermo-induced, growth-promoting gene expression levels in the seedlings shown in **j**. Seedlings were grown at 20 °C and then transferred to 27 °C or maintained at 20 °C for 6 hours under Rc. Data was shown from three biological replicates. Different letters denote significant statistical differences (one-way ANOVA, n = 3, p < 0.05).

**Supplementary Fig. 7 Characterizations of *PIF4* transgenic lines in the *pifq* mutant background. a**, Hypocotyl length measurements of seedlings. Wild-type (Col-0), *pifq* (*pif1 pif3 pif4 pif5* quadruple) mutant, and transgenic lines expressing *PIF4*-*mGFPmut2*-^*6*^*His* or *PIF4*^*mTAD*^-*mGFPmut2*-^*6*^*His* driven by the *PIF4* native promoter in the *pifq* mutant background were grown for 4 days in Rc (50 μmol m^−2^ s^−1^). The white and grey boxes represent hypocotyl length at 20 °C and 27 °C, respectively. The elements of box plots are as follows: center line, median; box limits, first and third quartiles; whiskers, minimum and maximum values; points, all data points. Different letters denote significant statistical differences between the absolute hypocotyl length of each line (two-way ANOVA, n ≥ 13, p < 0.05). **b**, Relationship between *PIF4* transcript level and hypocotyl length at warm temperatures. The hypocotyl length of each genotype grown at 27 °C in a was plotted against the relative *PIF4* transcript level measured in the same condition (that in Col-0 was set to 1). Nonlinear regression (sigmoidal, 4PL) was performed separately for *PIF4*-*mGFPmut2*-^*6*^*His/pifq* (blue) and *PIF4*^*mTAD*^-*mGFPmut2*-^*6*^*His/pifq* lines (yellow).

Supplementary Table 1. Primers used for plasmid constructions.

Supplementary Table 2. RT-qPCR primers used in this study.

## Data Availability

The original contributions presented in the study are included in the article/Supplementary Material; further inquiries can be directed to the corresponding author.
